# Supplementary material for: Mechanistic and evolutionary insights into isoform-specific ‘supercharging’ in DCLK family kinases
Source: bioRxiv. 2023 Jul 18:2023.03.29.534689. Originally published 2023 Mar 29. Preprint. [Version 2] doi: 10.1101/2023.03.29.534689 (PMC10081240; doi:10.1101/2023.03.29.534689)
Supplement: Supplement 1 [file NIHPP2023.03.29.534689v2-supplement-1.pdf]

bioRxiv preprint doi: https://doi.org/10.1101/2023.03.29.534689; this version posted July 18, 2023. The copyright holder for this preprint (which was not certified by peer review) is the author/funder, who has granted bioRxiv a license to display the preprint in perpetuity. It is made available under aCC-BY 4.0 International license.

Figure supplements for:

Mechanistic and evolutionary insights into isoform-specific 'supercharging' in DCLK family kinases

Aarya Venkat[1+], Grace Watterson[1+], Dominic P. Byrne[2+], Brady O'Boyle[1], Safal Shrestha[3], Nathan Gravel[3], Emma E. Fairweather[2], Leonard A. Daly[2,4], Claire Bunn[1], Wayland Yeung[3], Ishan Aggarwal[1], Samiksha Katiyar[1], Claire E. Eyers[2,4], Patrick A. Eyers[2*], and Natarajan Kannan[1,3*]

Affiliations:

[1]Department of Biochemistry and Molecular Biology, University of Georgia, Athens, GA 30602, USA
[2]Department of Biochemistry and Systems Biology, Institute of Systems, Molecular and Integrative Biology, University of Liverpool, Liverpool, L69 7ZB, UK
[3]Institute of Bioinformatics, University of Georgia, Athens, GA 30602, USA
[4]Centre for Proteome Research, Department of Biochemistry and Systems Biology, Institute of Systems, Molecular and Integrative Biology, University of Liverpool, Liverpool, L69 7ZB, UK

+ equal contributions

*Correspondence to: Natarajan Kannan, Email: nkannan@uga.edu or Patrick Eyers, Email: patrick.eyers@liverpool.ac.uk

bioRxiv preprint doi: https://doi.org/10.1101/2023.03.29.534689; this version posted July 18, 2023. The copyright holder for this preprint (which was not certified by peer review) is the author/funder, who has granted bioRxiv a license to display the preprint in perpetuity. It is made available under aCC-BY 4.0 International license.

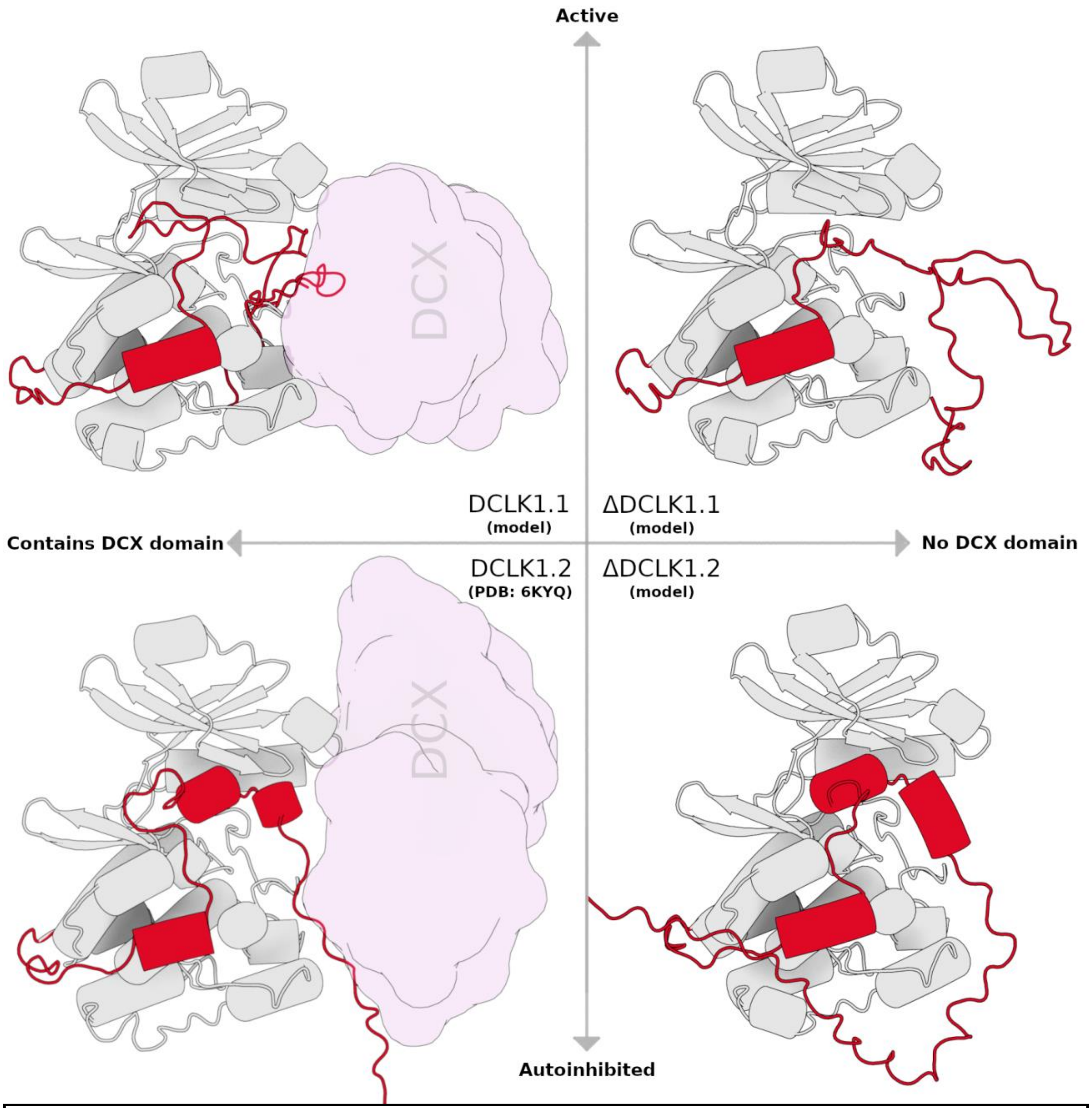

Figure 1-figure supplement 1: Structural cartoon depicting each DCLK1 isoform, categorized by the presence of DCX domain and related to enzymatic activity.

bioRxiv preprint doi: https://doi.org/10.1101/2023.03.29.534689; this version posted July 18, 2023. The copyright holder for this preprint (which was not certified by peer review) is the author/funder, who has granted bioRxiv a license to display the preprint in perpetuity. It is made available under aCC-BY 4.0 International license.

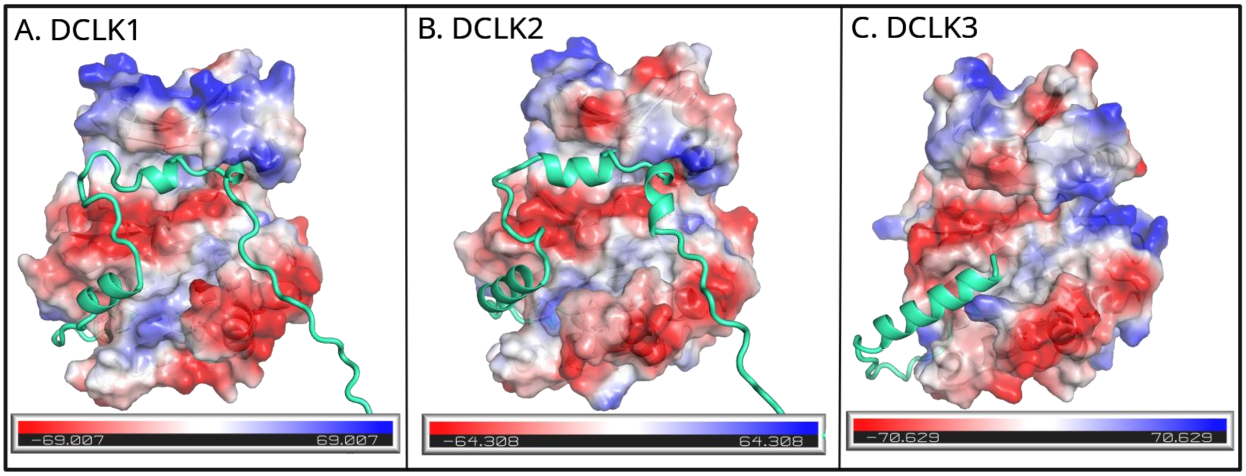

Figure 2-figure supplement 1: Electrostatic surface views of full-length DCLK paralog (DCLK1, DCLK2, and DCLK3) in the same orientation. These structures show how the tail packs against the substrate binding pocket of the kinase domain in each paralog. The electrostatic surface is color-coded with negative (red) and positive (blue) charges. Similarities in the distribution of charges among paralogs can influence substrate binding affinity and kinase activity.

bioRxiv preprint doi: https://doi.org/10.1101/2023.03.29.534689; this version posted July 18, 2023. The copyright holder for this preprint (which was not certified by peer review) is the author/funder, who has granted bioRxiv a license to display the preprint in perpetuity. It is made available under aCC-BY 4.0 International license.

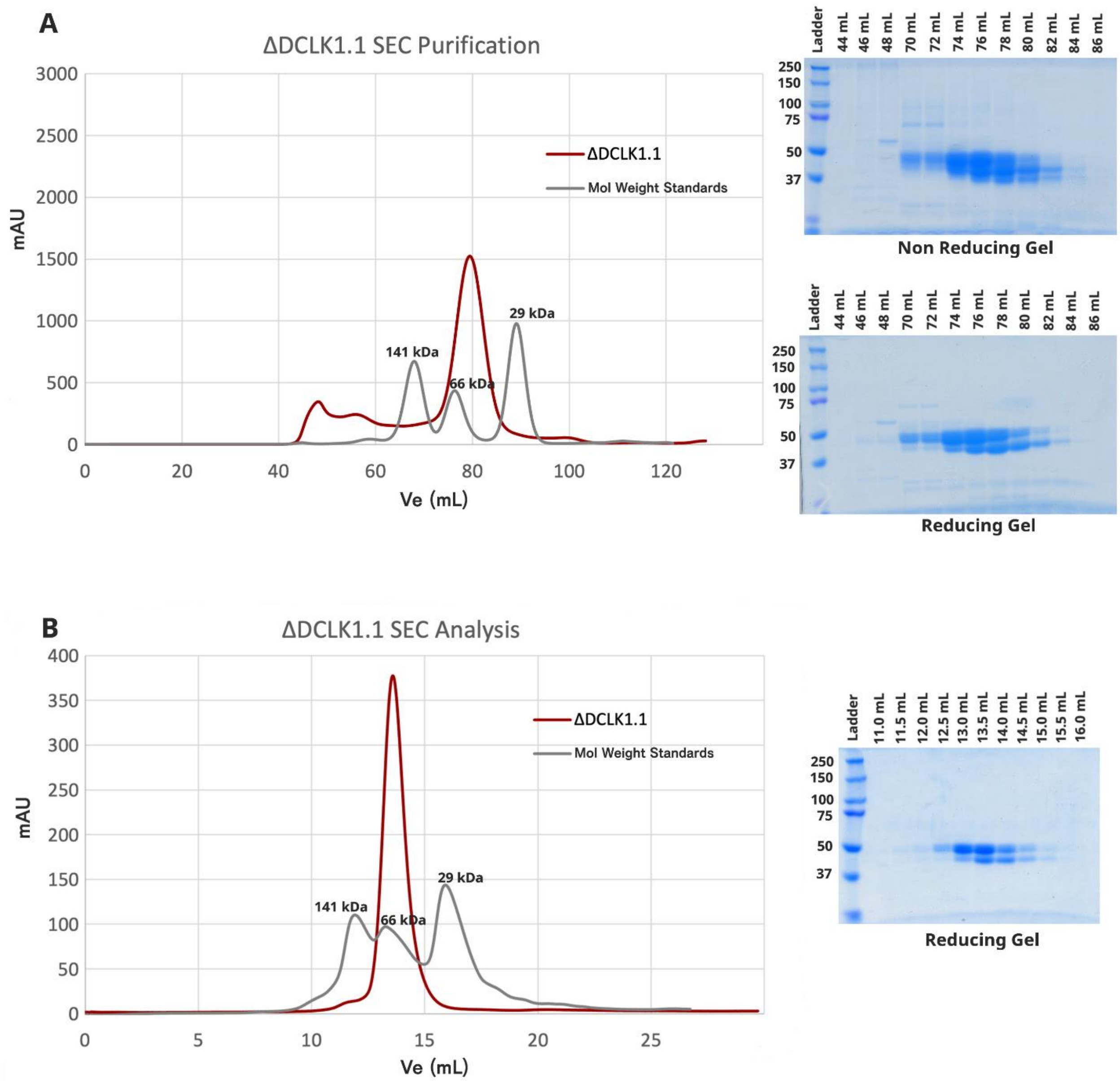

Figure 3-figure supplement 1: A) Purification of DCLK1.1$^{351-729}$ by SEC reveals a single peak, representing monomeric protein in solution. Reducing and non-reducing SDS-PAGE show the elution profiles of the purified species. **B)** Analytical SEC of purified DCLK1.1$^{351-729}$ (1.1 mg) confirms a single monomeric species.

bioRxiv preprint doi: https://doi.org/10.1101/2023.03.29.534689; this version posted July 18, 2023. The copyright holder for this preprint (which was not certified by peer review) is the author/funder, who has granted bioRxiv a license to display the preprint in perpetuity. It is made available under aCC-BY 4.0 International license.

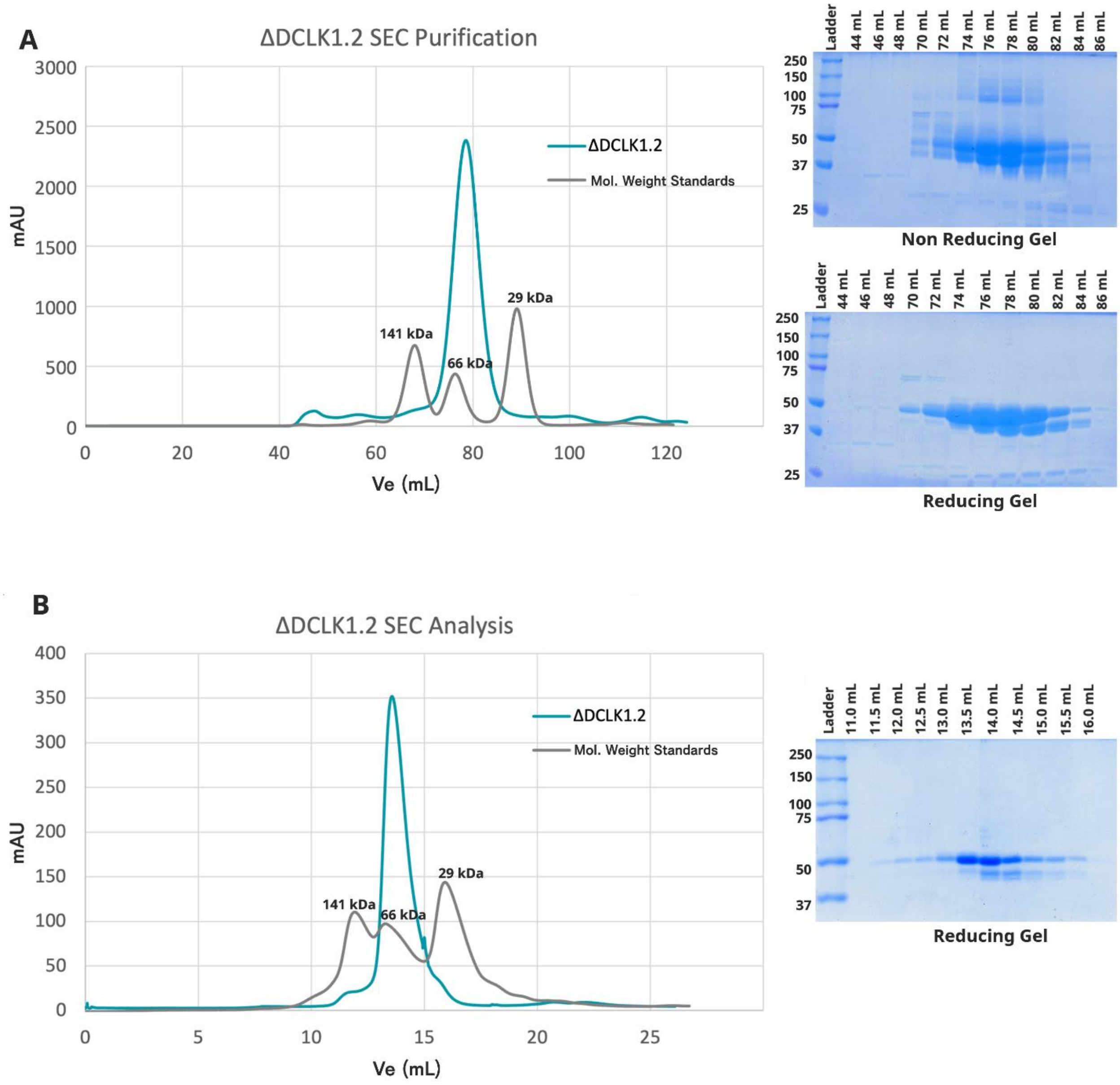

Figure 3-figure supplement 2: A) Purification of DCLK1.2$^{351\text{-}740}$ by SEC reveals a single peak, representing monomeric protein in solution. Reducing and non-reducing SDS-PAGE showing the elution profiles of the purified proteins. **B)** Analytical SEC of purified DCLK1.2$^{351\text{-}740}$ (1.1 mg) confirms a single monomeric species.

bioRxiv preprint doi: https://doi.org/10.1101/2023.03.29.534689; this version posted July 18, 2023. The copyright holder for this preprint (which was not certified by peer review) is the author/funder, who has granted bioRxiv a license to display the preprint in perpetuity. It is made available under aCC-BY 4.0 International license.

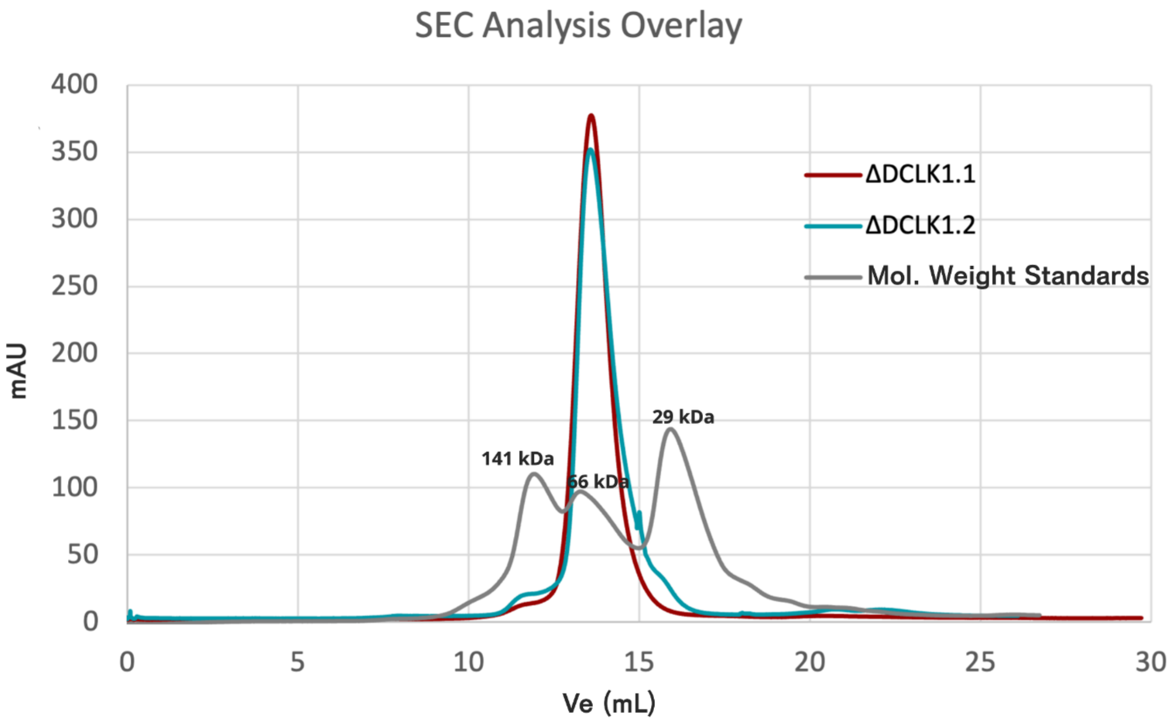

Figure 3-figure supplement 3: Analytical SEC of DCLK1.1$^{351-729}$ and DCLK1.2$^{351-740}$. Overlaid chromatograms showing elution profiles for both proteins and molecular weight standards. Both DCLK1 isoforms elute as single peaks at the predicted molecular weight for the monomeric protein.

bioRxiv preprint doi: https://doi.org/10.1101/2023.03.29.534689; this version posted July 18, 2023. The copyright holder for this preprint (which was not certified by peer review) is the author/funder, who has granted bioRxiv a license to display the preprint in perpetuity. It is made available under aCC-BY 4.0 International license.

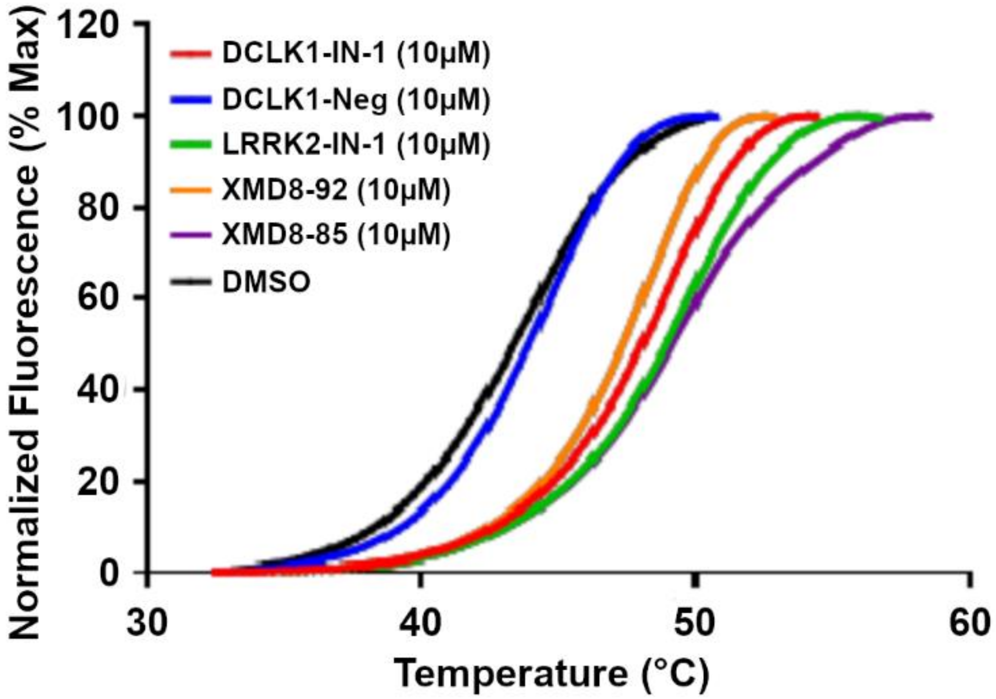

Figure 3-figure supplement 4: DSF profile of $DCLK1_{351-689}$ in the presence of DMSO or a panel of DCLK1 inhibitor compounds. DCLK1-Neg is a negative control.

bioRxiv preprint doi: https://doi.org/10.1101/2023.03.29.534689; this version posted July 18, 2023. The copyright holder for this preprint (which was not certified by peer review) is the author/funder, who has granted bioRxiv a license to display the preprint in perpetuity. It is made available under aCC-BY 4.0 International license.

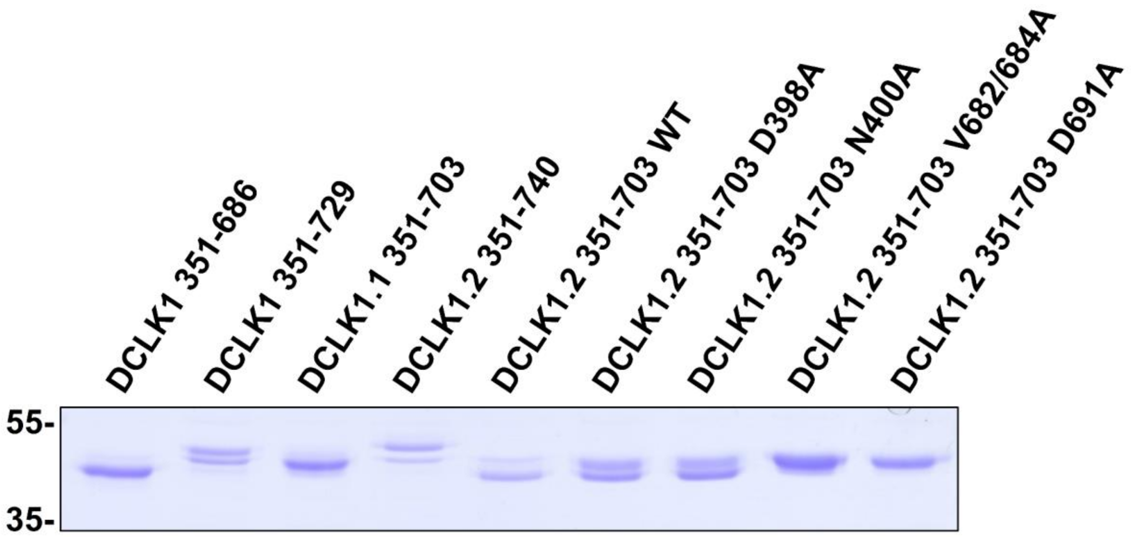

Figure 5-figure supplement 1: SDS-Page and Coomassie blue staining of each DCLK1 protein.

bioRxiv preprint doi: https://doi.org/10.1101/2023.03.29.534689; this version posted July 18, 2023. The copyright holder for this preprint (which was not certified by peer review) is the author/funder, who has granted bioRxiv a license to display the preprint in perpetuity. It is made available under aCC-BY 4.0 International license.

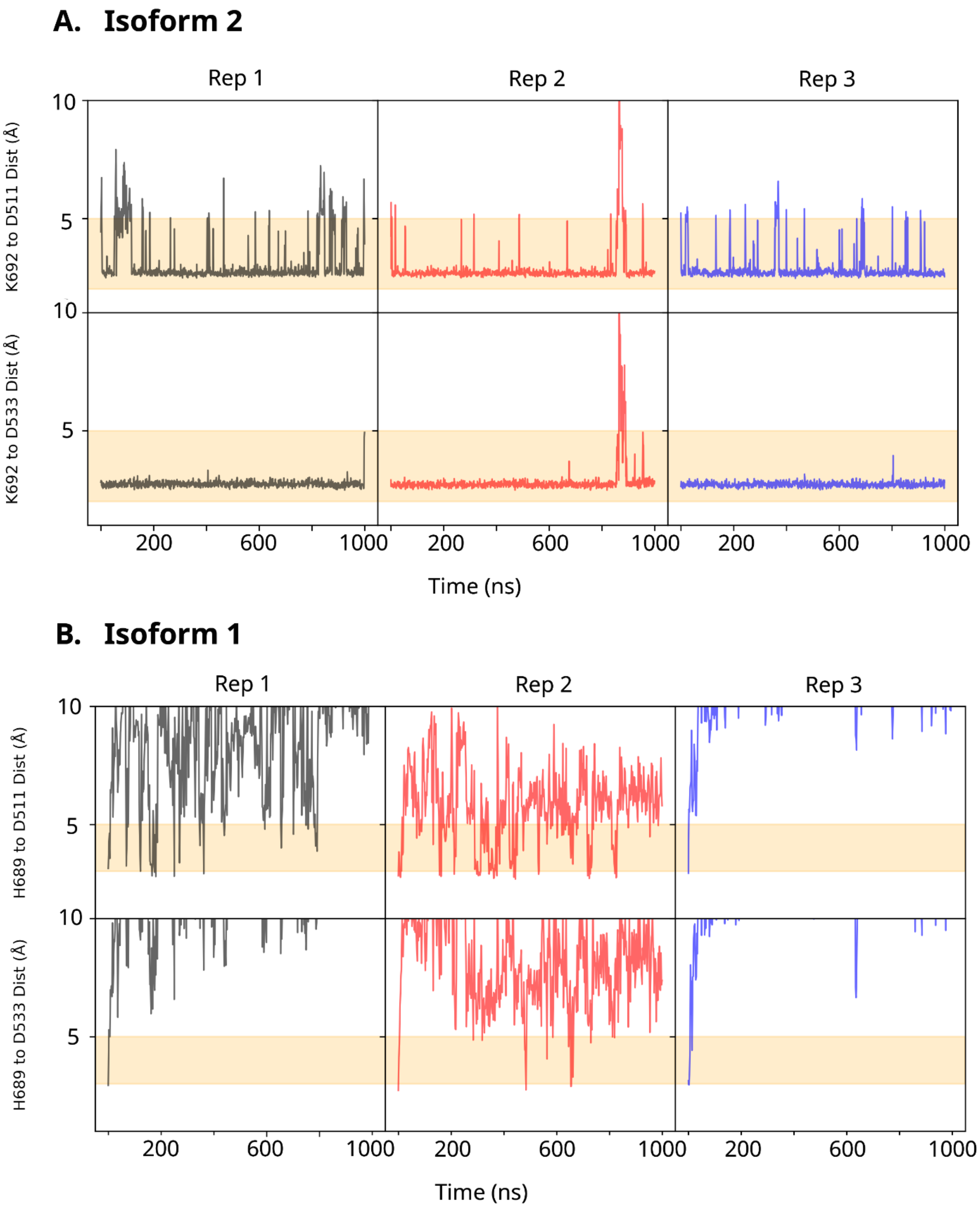

903

Figure 5-figure supplement 2: A) Minimum Distance of K692 in the DCLK1.2 C-tail forms significant stable interactions over microsecond replicates to the DFG and HRD aspartates. **B)** H689 in the DCLK1.1 C-tail, comparatively fails to interact with the DFG and HRD aspartates.

904

bioRxiv preprint doi: https://doi.org/10.1101/2023.03.29.534689; this version posted July 18, 2023. The copyright holder for this preprint (which was not certified by peer review) is the author/funder, who has granted bioRxiv a license to display the preprint in perpetuity. It is made available under aCC-BY 4.0 International license.

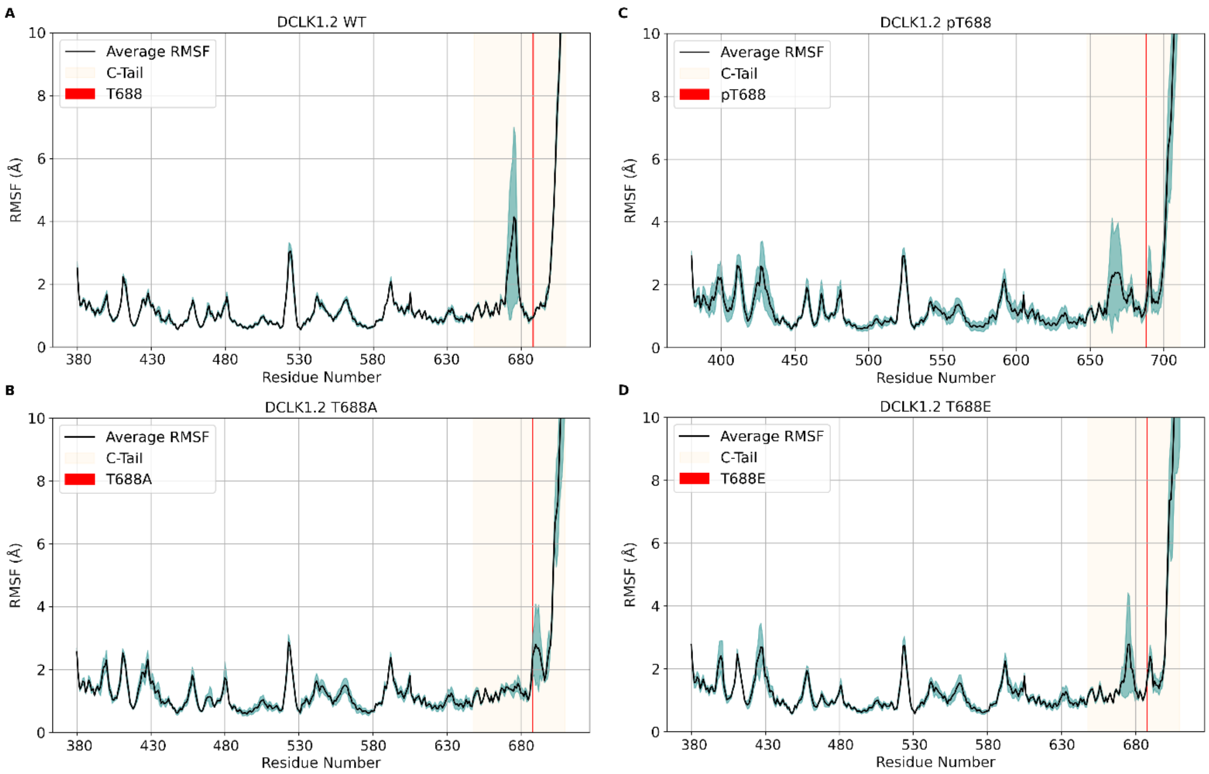

905

Figure 5-figure supplement 3: RMSF plots of MD simulations of ΔDCLK1.2 wt, pT688, T688A, and T688E, where T688 is demarcated by a red line and the entire ΔDCLK1.2 C-tail is highlighted in light yellow. The black line represents the average RMSF between three 500ns replicates and the blue shading represents standard deviation of the replicates, where less shading indicates convergence between replicates and increased shading indicates deviation.

906

bioRxiv preprint doi: https://doi.org/10.1101/2023.03.29.534689; this version posted July 18, 2023. The copyright holder for this preprint (which was not certified by peer review) is the author/funder, who has granted bioRxiv a license to display the preprint in perpetuity. It is made available under aCC-BY 4.0 International license.

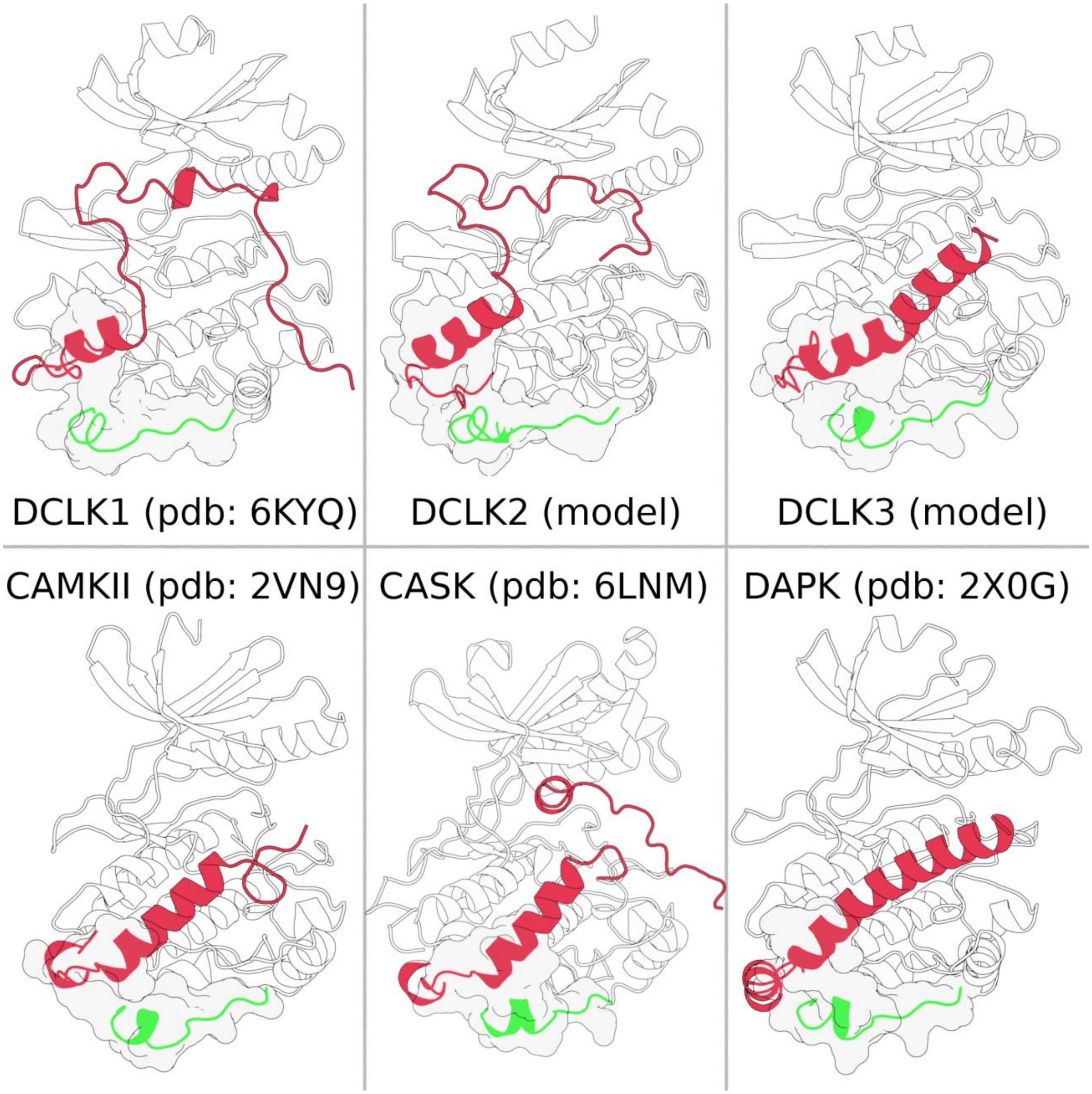

Figure 6-figure supplement 1: CAMK-specific insert (green) consistently making structural contacts (shown in surface representation) with the C-tail (red) across multiple CAMK families.

bioRxiv preprint doi: https://doi.org/10.1101/2023.03.29.534689; this version posted July 18, 2023. The copyright holder for this preprint (which was not certified by peer review) is the author/funder, who has granted bioRxiv a license to display the preprint in perpetuity. It is made available under aCC-BY 4.0 International license.

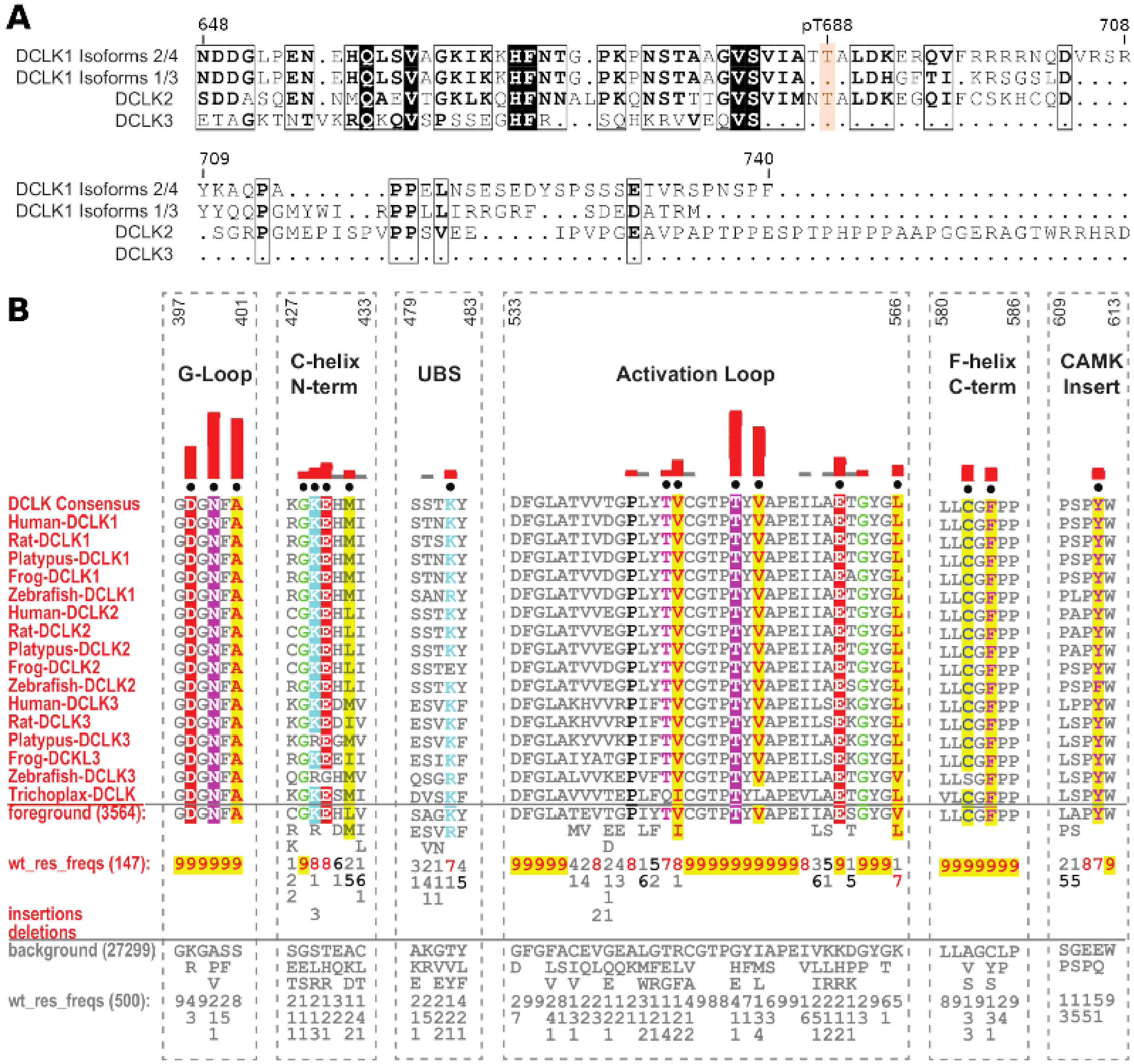

Figure 6-figure supplement 2: Identification of DCLK family-specific constraints. A) Sequence alignment of human DCLK paralogs including long and short isoforms of DCLK1. **B)** Sequence constraints that distinguish DCLK1/2/3 sequences from closely related CAMK sequences are shown in a contrast hierarchical alignment (CHA). The CHA shows DCLK1/2/3 sequences from diverse organisms as the display alignment. The foreground consists of 3564 DCLK sequences while the background alignment contains 27,299 related CAMK sequences. The foreground and background alignments are shown as residue frequencies below the display alignment in integer tenths (1–9). The histogram (red) indicates the extent to which distinguishing residues in the foreground diverge from the corresponding position in the background alignment. Black dots indicate the alignment positions used by the BPPS (Neuwald, 2014) procedure when classifying DCLK sequences from related CAMK sequences. Alignment number is based on the human DCLK1 sequence (Uniprot ID: O15075-1).

bioRxiv preprint doi: https://doi.org/10.1101/2023.03.29.534689; this version posted July 18, 2023. The copyright holder for this preprint (which was not certified by peer review) is the author/funder, who has granted bioRxiv a license to display the preprint in perpetuity. It is made available under aCC-BY 4.0 International license.

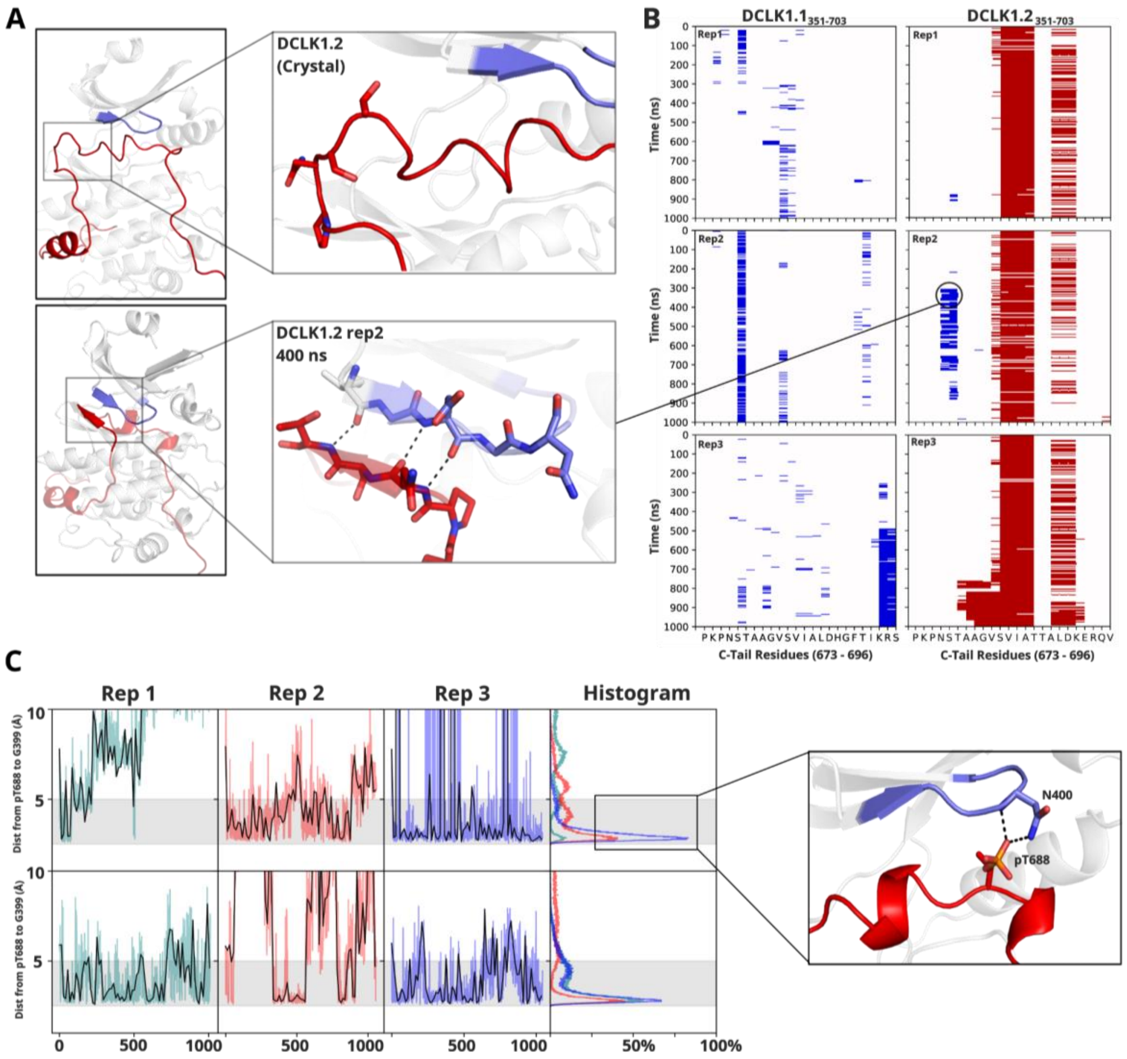

Figure 7-figure supplement 1: Molecular Dynamics of DCLK1 isoforms. **A-B)** Microsecond MD replicates from DCLK1.1 and DCLK1.2, showing the DSSP output plotted for the C-tail, where red lines represent alpha helices and blue lines represent beta sheets. **C)** Distance plots from MD replicates of the phosphorylated threonine highlighting the contact distance between pT688 phosphate and G399 of the G-loop.

bioRxiv preprint doi: https://doi.org/10.1101/2023.03.29.534689; this version posted July 18, 2023. The copyright holder for this preprint (which was not certified by peer review) is the author/funder, who has granted bioRxiv a license to display the preprint in perpetuity. It is made available under aCC-BY 4.0 International license.

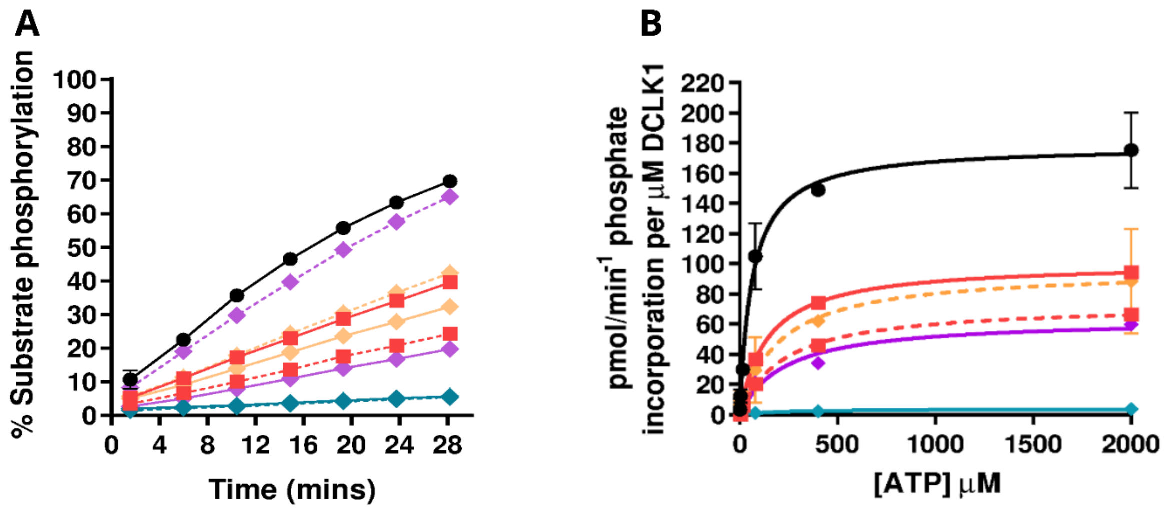

C

	DCLK1 351-686	DCLK1.1 351-703	DCLK1.1 351-729	DCLK1.2 351-703	DCLK1.2 D398A	DCLK1.2 V682/684T
**Vmax** pmol/min^{-1}	178.2 ±6.1	100.6 ±0.6	73.3 ±1.1	3.9 ±0.08	63.5 ±3.0	96.1 ±10.3
**Km** [ATP] µM	63.5 ±9.7	142.1 ±3.4	224 ±11.9	236.4 ±17.1	229.9 ±38.1	199.7 ±77.9

Figure 8-figure supplement 1: A) DCLK1 substrate phosphorylation (calculated as % total phosphopeptide) was quantified as a function of time for each of the indicated purified DCLK proteins in the presence of 1 mM ATP. Assays were performed side-by-side. Data is mean and SD from (N=4) independent experiments. **B)** Michaelis-Menten plots showing normalized DCLK1 activity in the presence of increasing concentrations of ATP, to tease apart effects of C-tail on ATP affinity. Data shown is mean and SD from (N=3) independent experiments. **C)** Table of calculated Vmax and Km [ATP] values obtained from (B).

bioRxiv preprint doi: https://doi.org/10.1101/2023.03.29.534689; this version posted July 18, 2023. The copyright holder for this preprint (which was not certified by peer review) is the author/funder, who has granted bioRxiv a license to display the preprint in perpetuity. It is made available under aCC-BY 4.0 International license.

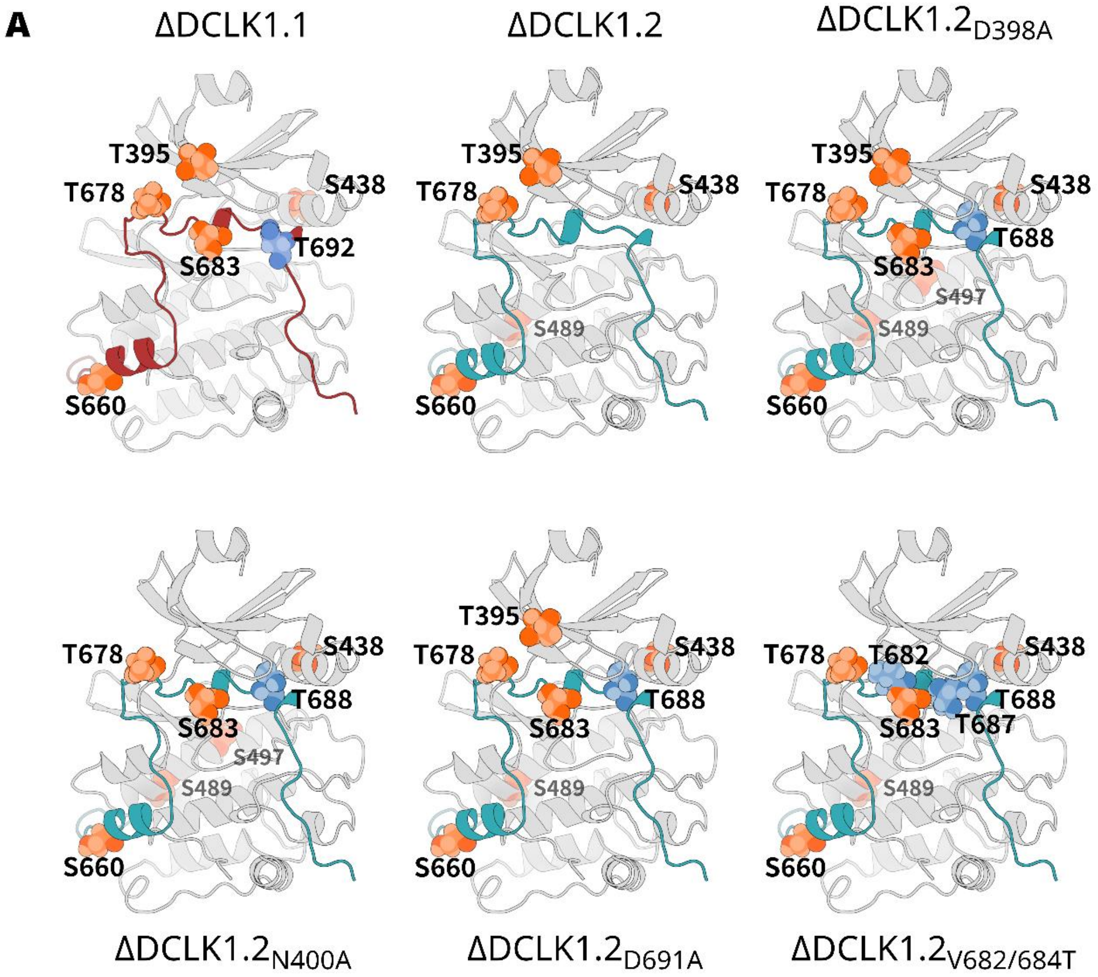

B

Sequence	Phosphosite in protein (ptmRS score)	MASCOT score	DCLK1.1 (351-703)	DLCK1.2 (351-703)
GKEHMIQNEVSILR	Ser 438 (100)	68	1.00	0.15
FSAVQVLEHPWVNDDGLPENEHQLSVAGK	Ser 660 (100)	42	1.00	0.01

C

Sequence	Phosphosite in protein (ptmRS score)	MASCOT score	Normalised fold change			
			WT	D398A	N400A	V682/684T
TIGDGNFAVVK	Thr 395 (100)	60	1.00	ND	ND	1.41
GKEHMIQNEVSILR	Ser 438 (100)	69	1.00	6.91	4.08	1.69
DASGMLYNLASAIK	Ser 489 (100)	75	1.00	1.24	0.42	0.07
FSAVQVLEHPWVNDDGLPENEHQLSVAGK	Ser 660 (100)	50	1.00	99.01	41.43	43.76

Figure 8-figure supplement 2: A) All mapped DCLK phosphorylation sites derived from LC-MS/MS analysis of DCLK1.1 and DCLK 1.2 proteins. Identified sites of phosphorylation at the kinase domain are colored in orange with isoform or mutant-specific phosphorylation sites colored in blue and mapped onto the structure of each protein **B)** Quantitative LC-MS/MS data showing tryptic phosphopeptides identified from DCLK1.1 and 1.2 that were directly comparable between isoforms. Detailed are peptide sequences, identified sites of phosphorylation (red), the site of phosphorylation within the protein polypeptide and the ptmRS score relevant to confidence of phosphosite localisation, as well as the Mascot score for peptide identification. Fold-changes in the relative abundance of the two phosphopeptides in DCLK 1.2 are computed with reference to these same two phosphopeptides in DCLK1.1, normalising against 3 non-modified peptides to account for potential difference in the amount analysed. **C)** As described in B, quantitative LC-MS/MS data for sites directly comparable between DCLK1.2 and its variants. Fold change in abundance could not be calculated for the peptide containing pThr 395 given the presence of the inserted amino acid mutations and the differences in relative ionisation efficiency for the resulting tryptic peptide.

bioRxiv preprint doi: https://doi.org/10.1101/2023.03.29.534689; this version posted July 18, 2023. The copyright holder for this preprint (which was not certified by peer review) is the author/funder, who has granted bioRxiv a license to display the preprint in perpetuity. It is made available under aCC-BY 4.0 International license.

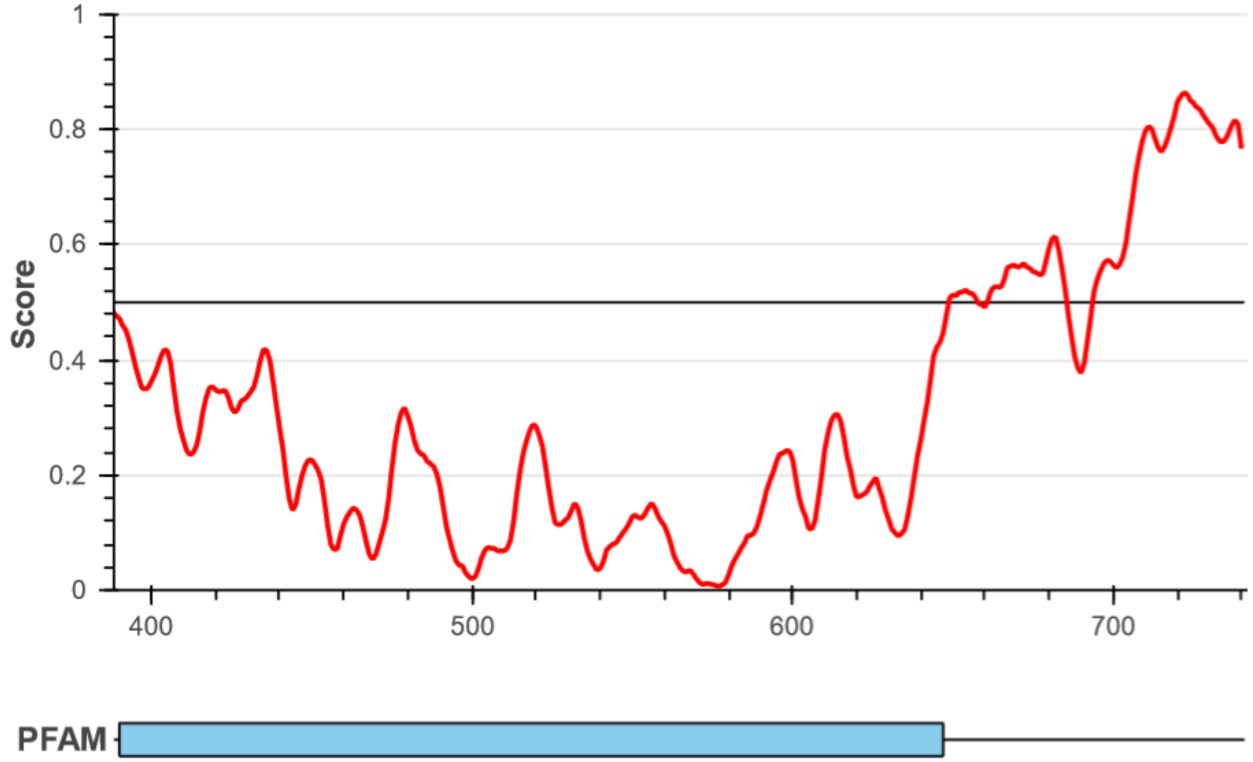

Figure 9-figure supplement 1: Intrinsic Disorder prediction of DCLK1.2 C-tail using IUPRED3.
