# Supplementary material for: Mechanistic and evolutionary insights into isoform-specific ‘supercharging’ in DCLK family kinases
Source: bioRxiv. 2023 Mar 29:2023.03.29.534689. Preprint. [Version 1] doi: 10.1101/2023.03.29.534689 (PMC10081240; doi:10.1101/2023.03.29.534689)

bioRxiv preprint doi: https://doi.org/10.1101/2023.03.29.534689; this version posted March 29, 2023. The copyright holder for this preprint (which was not certified by peer review) is the author/funder, who has granted bioRxiv a license to display the preprint in perpetuity. It is made available under aCC-BY 4.0 International license.

Figure supplements for:

Mechanistic and evolutionary insights into isoform-specific 'supercharging' in DCLK family kinases

Aarya Venkat[1+], Grace Watterson[1+], Dominic P. Byrne[2+], Brady O'Boyle[1], Safal Shrestha[3], Nathan Gravel[3], Emma E. Fairweather[2], Leonard A. Daly[2,4], Claire Bunn[1], Wayland Yeung[3], Ishan Aggarwal[1], Samiksha Katiyar[1], Claire E. Eyers[2,4], Patrick A. Eyers[2*], and Natarajan Kannan[1,3*]

Affiliations:

[1]Department of Biochemistry and Molecular Biology, University of Georgia, Athens, GA 30602, USA
[2]Department of Biochemistry and Systems Biology, Institute of Systems, Molecular and Integrative Biology, University of Liverpool, Liverpool, L69 7ZB, UK
[3]Institute of Bioinformatics, University of Georgia, Athens, GA 30602, USA
[4]Centre for Proteome Research, Department of Biochemistry and Systems Biology, Institute of Systems, Molecular and Integrative Biology, University of Liverpool, Liverpool, L69 7ZB, UK

+ equal contributions

*Correspondence to: Natarajan Kannan, Email: nkannan@uga.edu or Patrick Eyers, Email: patrick.eyers@liverpool.ac.uk

bioRxiv preprint doi: https://doi.org/10.1101/2023.03.29.534689; this version posted March 29, 2023. The copyright holder for this preprint (which was not certified by peer review) is the author/funder, who has granted bioRxiv a license to display the preprint in perpetuity. It is made available under aCC-BY 4.0 International license.

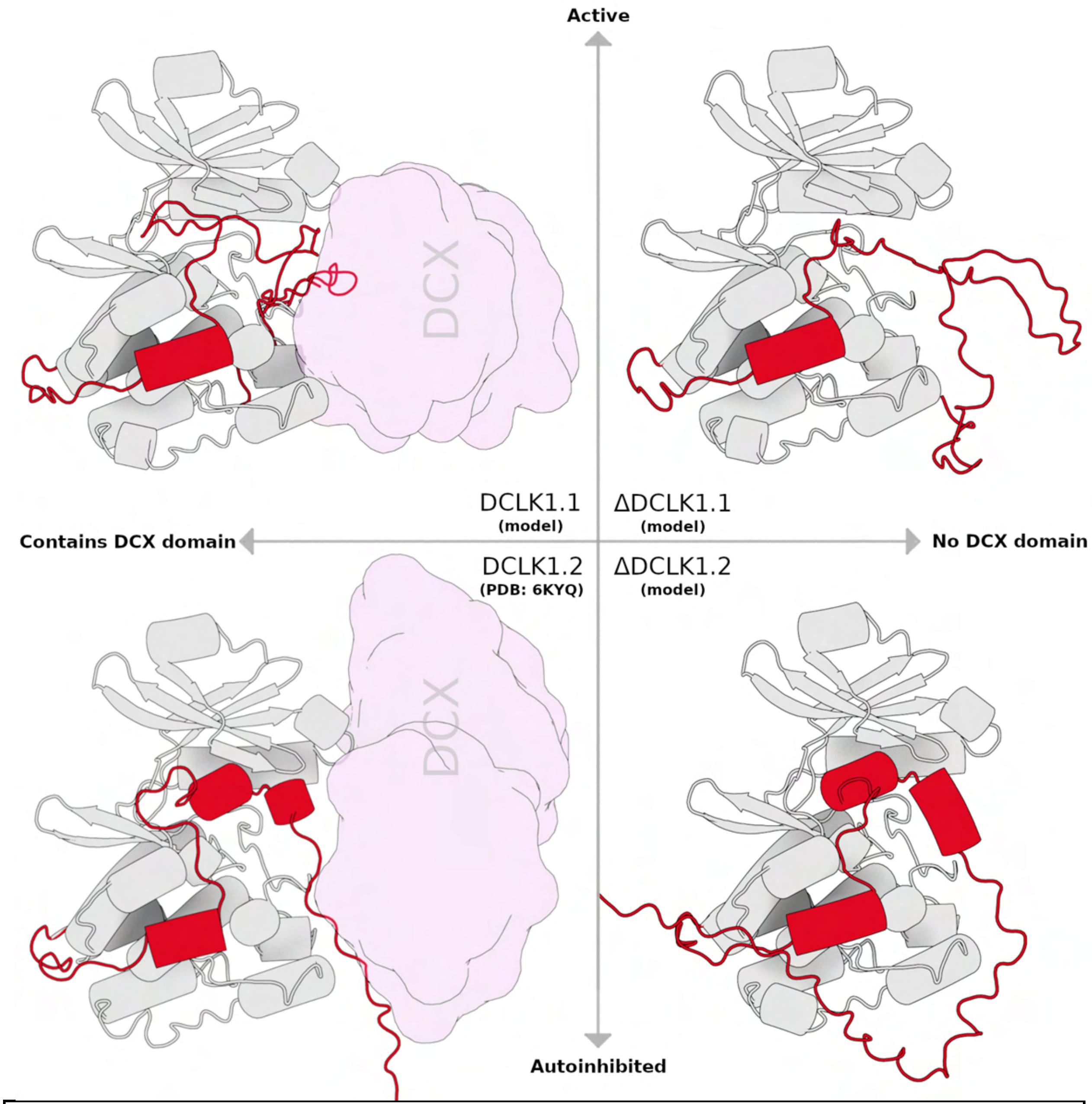

846

Figure 1-figure supplement 1: Structural cartoon depicting each DCLK1 isoform, categorizing them by presence of DCX domain and enzymatic activity.

847
848

bioRxiv preprint doi: https://doi.org/10.1101/2023.03.29.534689; this version posted March 29, 2023. The copyright holder for this preprint (which was not certified by peer review) is the author/funder, who has granted bioRxiv a license to display the preprint in perpetuity. It is made available under aCC-BY 4.0 International license.

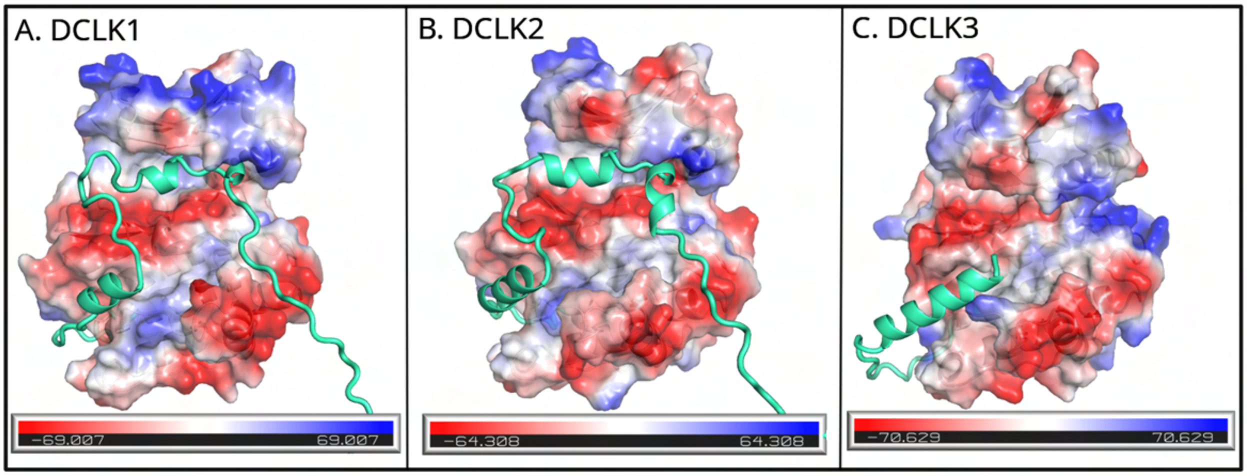

Figure 2-figure supplement 1: Each of the full-length DCLK paralogs showing how the tail packs against the substrate binding pocket of the kinase domain.

bioRxiv preprint doi: https://doi.org/10.1101/2023.03.29.534689; this version posted March 29, 2023. The copyright holder for this preprint (which was not certified by peer review) is the author/funder, who has granted bioRxiv a license to display the preprint in perpetuity. It is made available under aCC-BY 4.0 International license.

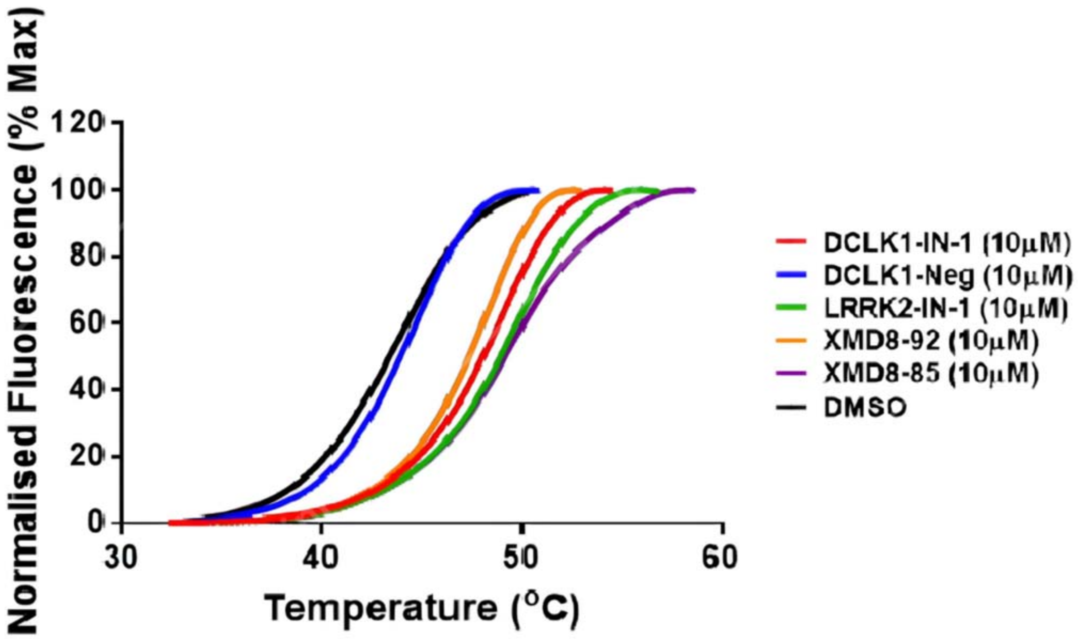

Figure 3-figure supplement 1: Fluorescence for DCLK1$_{cat}$ in presence of DMSO and different inhibitor conditions.

bioRxiv preprint doi: https://doi.org/10.1101/2023.03.29.534689; this version posted March 29, 2023. The copyright holder for this preprint (which was not certified by peer review) is the author/funder, who has granted bioRxiv a license to display the preprint in perpetuity. It is made available under aCC-BY 4.0 International license.

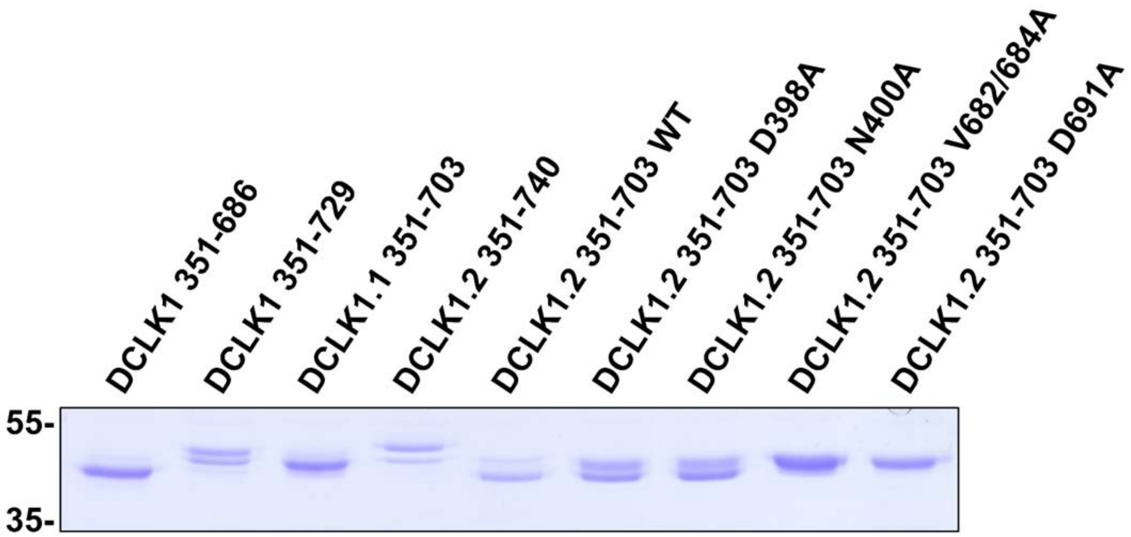

Figure 5-figure supplement 1: SDS-Page and Coomassie blue staining of each DCLK1 construct.

bioRxiv preprint doi: https://doi.org/10.1101/2023.03.29.534689; this version posted March 29, 2023. The copyright holder for this preprint (which was not certified by peer review) is the author/funder, who has granted bioRxiv a license to display the preprint in perpetuity. It is made available under aCC-BY 4.0 International license.

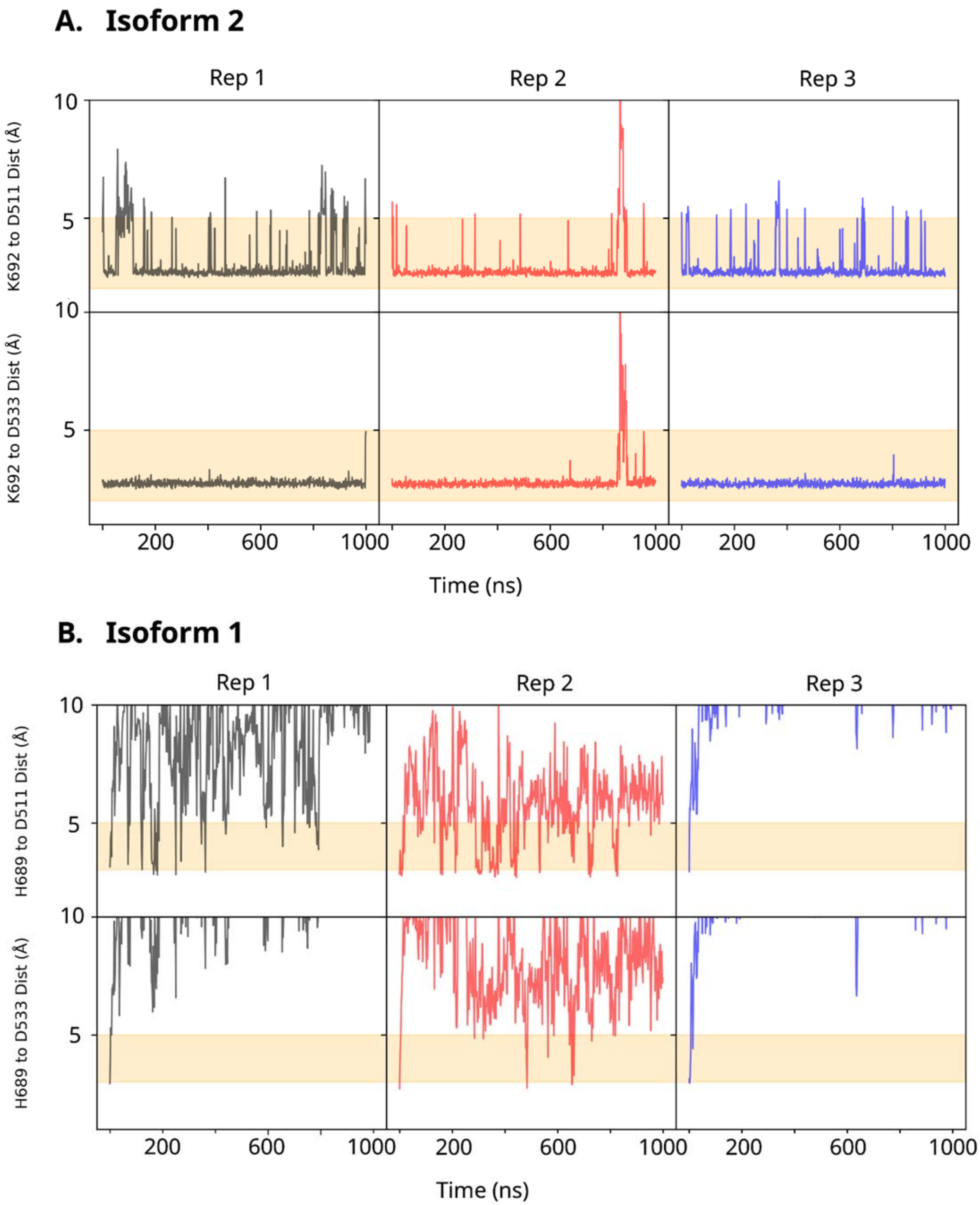

858

Figure 5-figure supplement 2: A) Minimum Distance of K692 in the DCLK1.2 C-tail forms significant stable interactions over microsecond replicates to the DFG and HRD aspartates. **B)** H689 in the DCLK1.1 C-tail, comparatively fails to interact with the DFG and HRD aspartates.

859

bioRxiv preprint doi: https://doi.org/10.1101/2023.03.29.534689; this version posted March 29, 2023. The copyright holder for this preprint (which was not certified by peer review) is the author/funder, who has granted bioRxiv a license to display the preprint in perpetuity. It is made available under aCC-BY 4.0 International license.

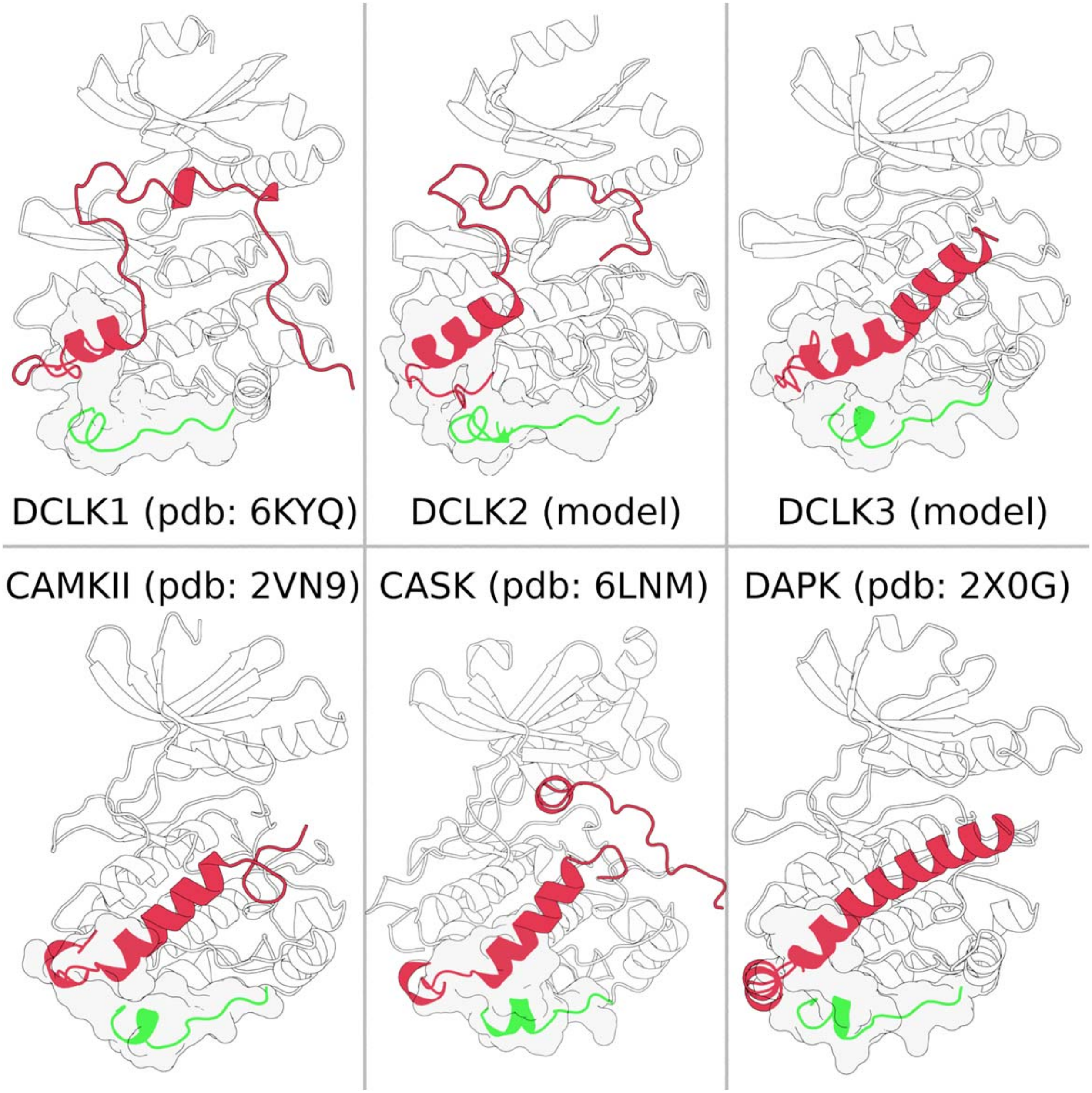

Figure 6-figure supplement 1: CAMK-specific insert (green) consistently making structural contacts (shown in surface representation) with the C-tail (red) across multiple CAMK families.

bioRxiv preprint doi: https://doi.org/10.1101/2023.03.29.534689; this version posted March 29, 2023. The copyright holder for this preprint (which was not certified by peer review) is the author/funder, who has granted bioRxiv a license to display the preprint in perpetuity. It is made available under aCC-BY 4.0 International license.

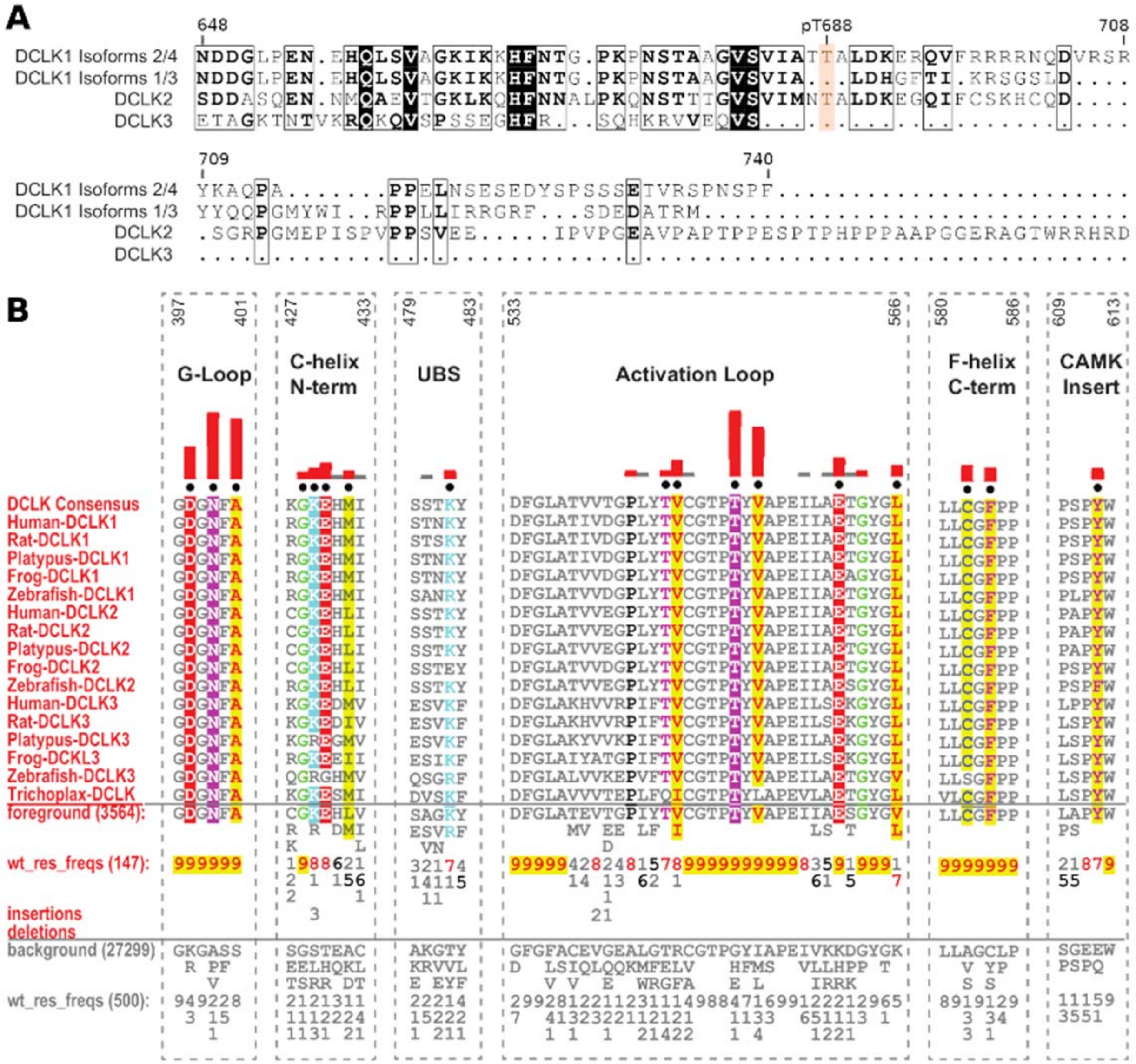

Figure 6-figure supplement 2: Identification of DCLK family-specific constraints. A) Sequence alignment of human DCLK paralogs including long and short isoforms of DCLK1. **B)** Sequence constraints that distinguish DCLK1/2/3 sequences from closely related CAMK sequences are shown in a contrast hierarchical alignment (CHA). The CHA shows DCLK1/2/3 sequences from diverse organisms as the display alignment. The foreground consists of 3564 DCLK sequences while the background alignment contains 27,299 related CAMK sequences. The foreground and background alignments are shown as residue frequencies below the display alignment in integer tenths (1–9). The histogram (red) indicates the extent to which distinguishing residues in the foreground diverge from the corresponding position in the background alignment. Black dots indicate the alignment positions used by the BPPS (Neuwald, 2014) procedure when classifying DCLK sequences from related CAMK sequences. Alignment number is based on the human DCLK1 sequence (Uniprot ID: O15075-1).

bioRxiv preprint doi: https://doi.org/10.1101/2023.03.29.534689; this version posted March 29, 2023. The copyright holder for this preprint (which was not certified by peer review) is the author/funder, who has granted bioRxiv a license to display the preprint in perpetuity. It is made available under aCC-BY 4.0 International license.

866

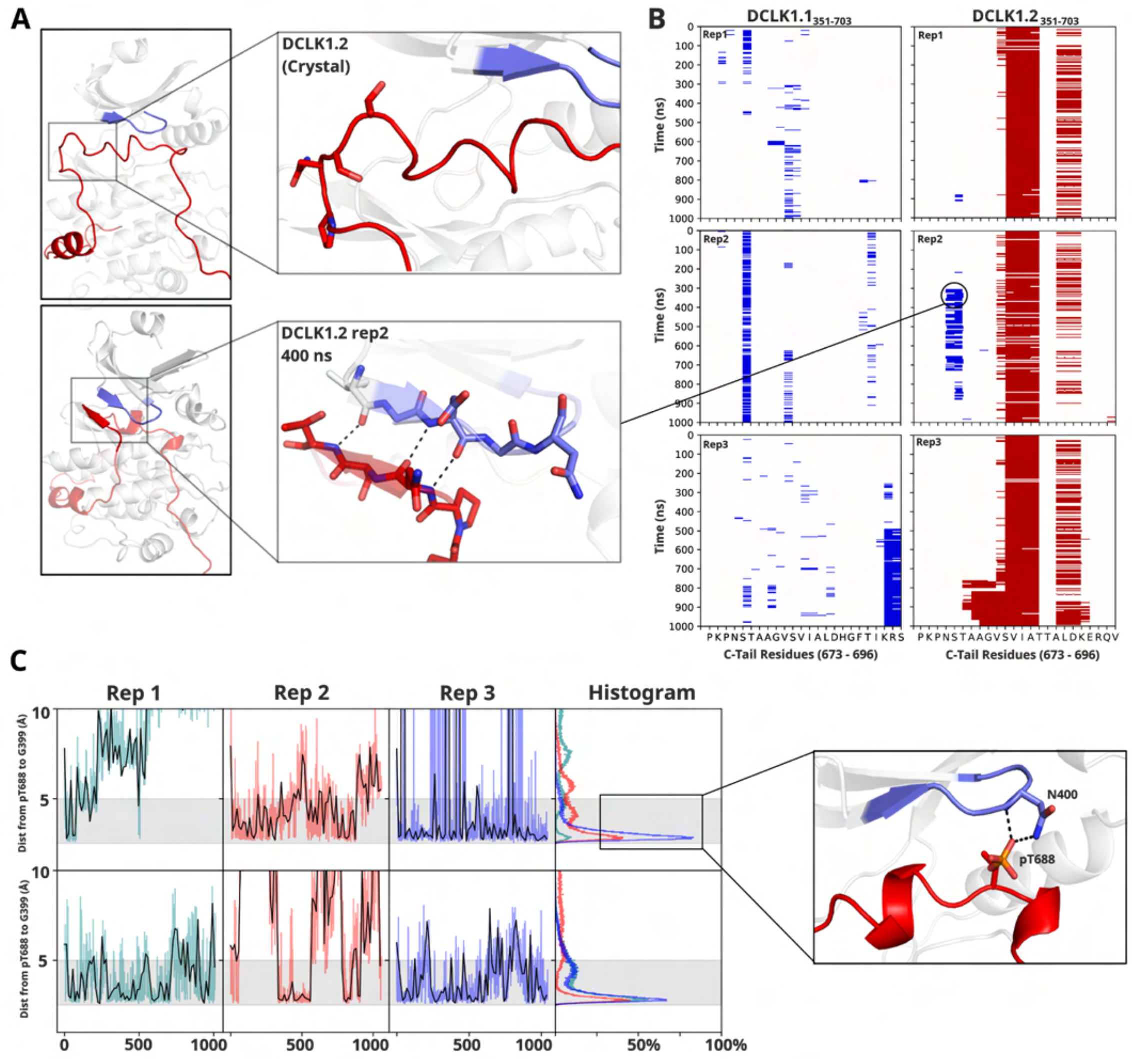

867

Figure 7-figure supplement 1: Molecular Dynamics of DCLK1 isoforms. **A-B)** Microsecond MD replicates from DCLK1.1 and DCLK1.2, showing the DSSP output plotted for the C-tail, where red lines represent alpha helices and blue lines represent beta sheets. **C)** Distance plots from MD replicates of the phosphorylated threonine highlighting the contact distance between pT688 phosphate and G399 of the G-loop.

868

bioRxiv preprint doi: https://doi.org/10.1101/2023.03.29.534689; this version posted March 29, 2023. The copyright holder for this preprint (which was not certified by peer review) is the author/funder, who has granted bioRxiv a license to display the preprint in perpetuity. It is made available under aCC-BY 4.0 International license.

A

Sequence	Phosphosite in protein (ptmRS score)	MASCOT score	DCLK1.1 (351-703)	DLCK1.2 (351-703)
GKEHMIQNEVSILR	Ser 438 (100)	68	1.00	0.15
FSAVQVLEHPWVNDDGLPENEHQLSVAGK	Ser 660 (100)	42	1.00	0.01

B

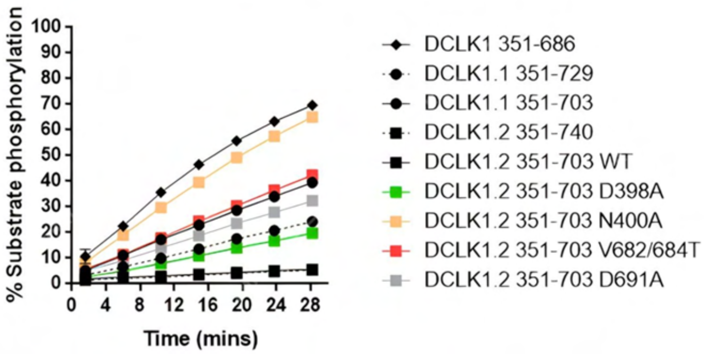

C

Sequence	Phosphosite in protein (ptmRS score)	MASCOT score	Normalised fold change			
			WT	D398A	N400A	V682/684T
TIGDGNFAVVK	Thr 395 (100)	60	1.00	ND	ND	1.41
GKEHMIQNEVSILR	Ser 438 (100)	69	1.00	6.91	4.08	1.69
DASGMLYNLASAIK	Ser 489 (100)	75	1.00	1.24	0.42	0.07
FSAVQVLEHPWVNDDGLPENEHQLSVAGK	Ser 660 (100)	50	1.00	99.01	41.43	43.76

Figure 8-figure supplement 1: A) Quantitative LC-MS/MS data showing tryptic phosphopeptides identified from DCLK1.1 and 1.2. Detailed are peptide sequences, identified sites of phosphorylation (red), the site of phosphorylation within the protein and the ptmRS score relevant to confidence of phosphosite localisation, as well as the Mascot score for peptide identification. Fold change in the relative abundance of the two phosphopeptides in DCLK 1.2 is computed with reference to these same two phosphopeptides in DCLK1.1., normalising against 3 non-modified peptides to account for potential difference in the amount analysed. **B**) DCLK1 substrate phosphorylation (reported as % total phosphopeptide in reaction) was quantified as a function of time for each of the indicated purified DCLK proteins. Assays were performed side-by-side in quadriplicate. **C)** As described in A, except fold change in abundance could not be calculated for the peptide containing pThr 395 given the presence of the inserted mutations and the potential differences in relative ionisation efficiency for the resulting tryptic peptide.

bioRxiv preprint doi: https://doi.org/10.1101/2023.03.29.534689; this version posted March 29, 2023. The copyright holder for this preprint (which was not certified by peer review) is the author/funder, who has granted bioRxiv a license to display the preprint in perpetuity. It is made available under aCC-BY 4.0 International license.

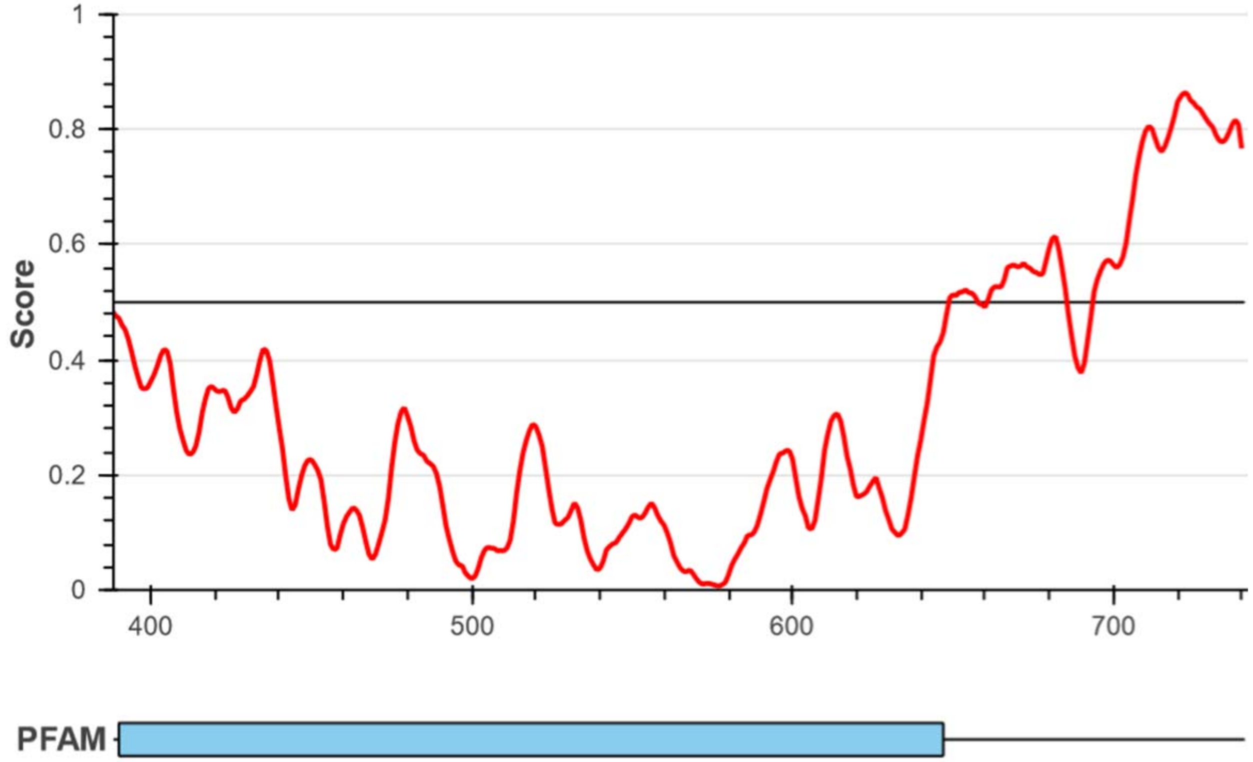

Figure 9-figure supplement 1: Intrinsic Disorder prediction of DCLK1.2 C-tail using IUPRED3.

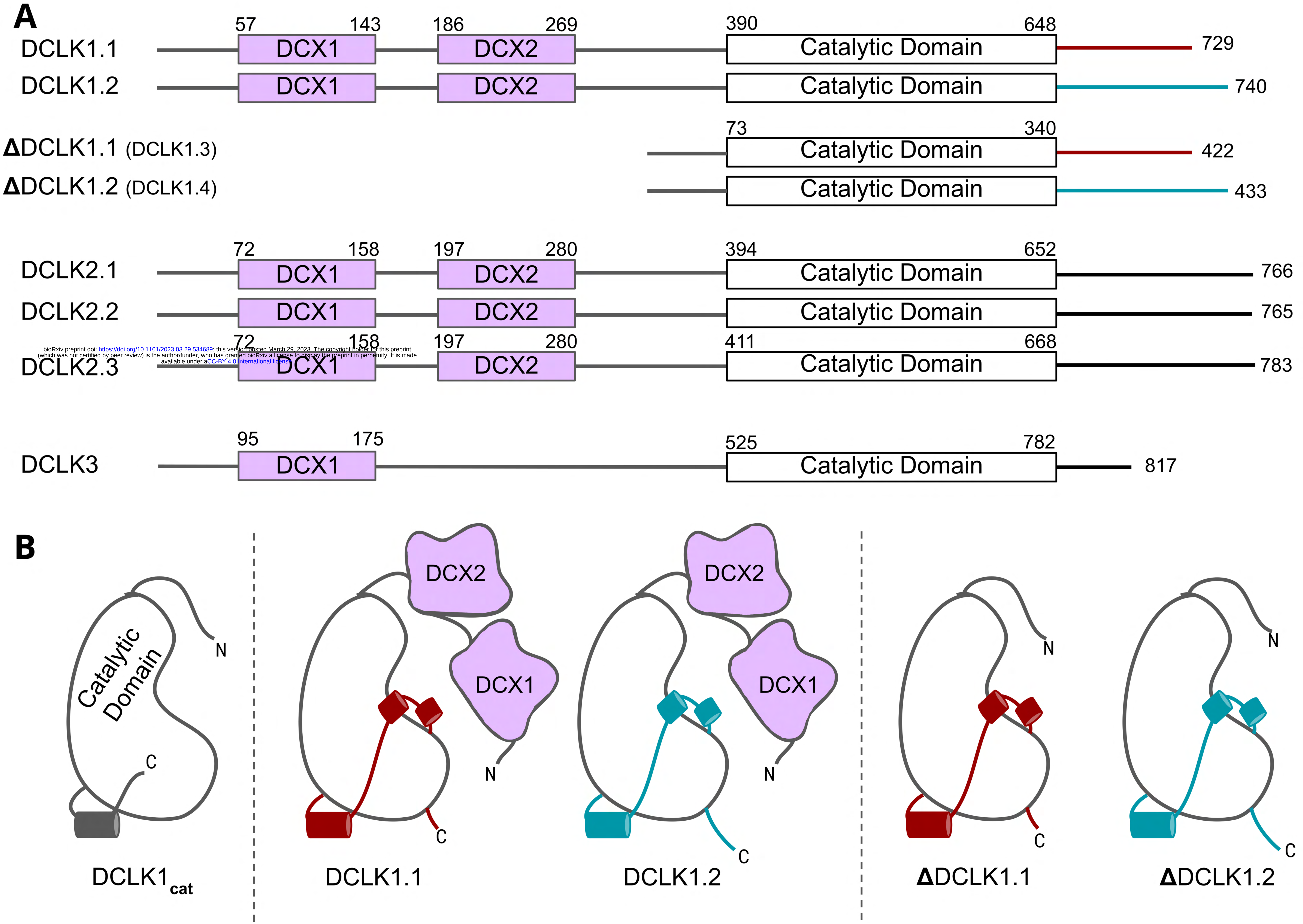

bioRxiv preprint doi: https://doi.org/10.1101/2023.03.29.534689; this version posted March 29, 2023. The copyright holder for this preprint (which was not certified by peer review) is the author/funder, who has granted bioRxiv a license to display the preprint in perpetuity. It is made available under aCC-BY 4.0 International license.

bioRxiv preprint doi: https://doi.org/10.1101/2023.03.29.534689; this version posted March 29, 2023. The copyright holder for this preprint (which was not certified by peer review) is the author/funder, who has granted bioRxiv a license to display the preprint in perpetuity. It is made available under aCC-BY 4.0 International license.

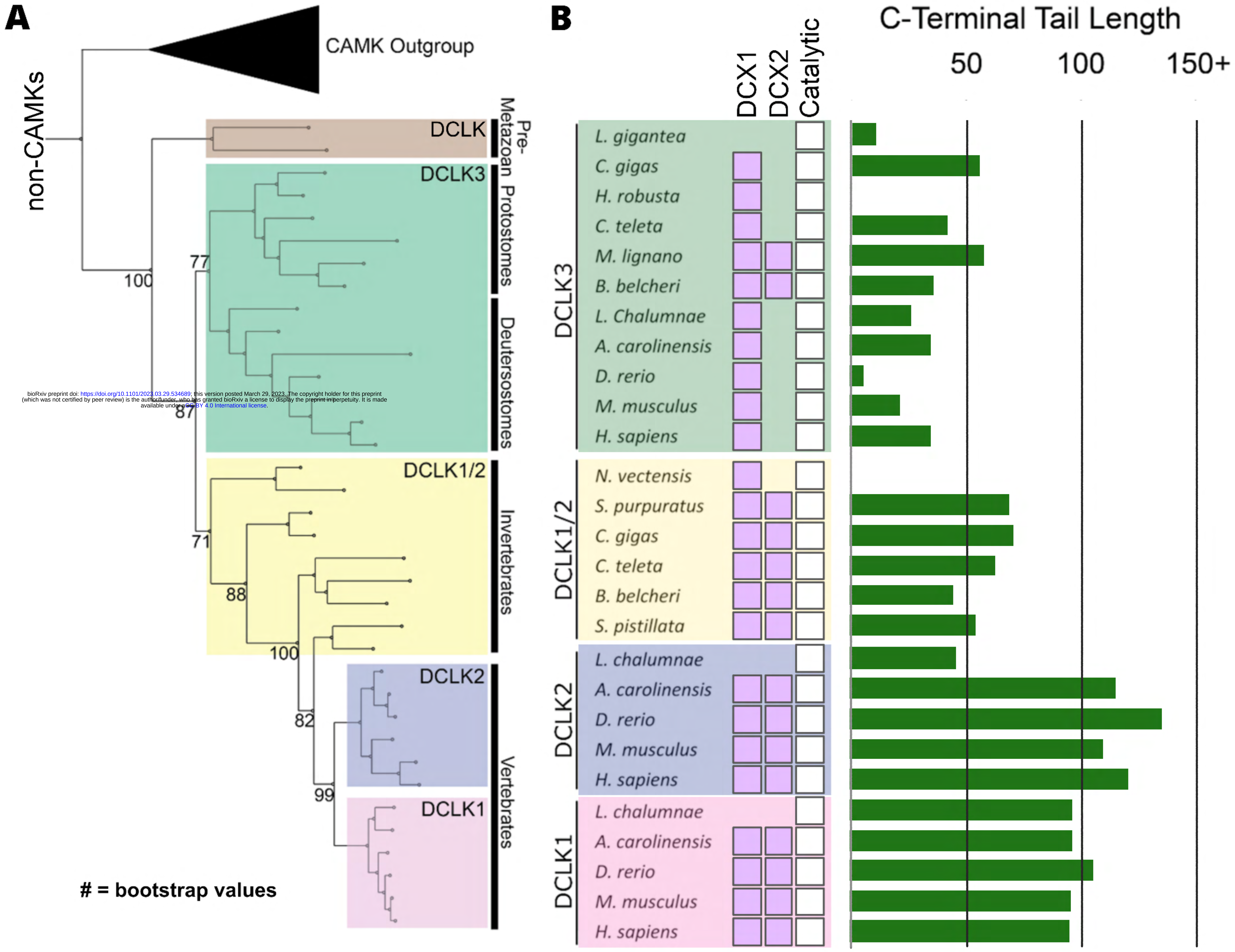

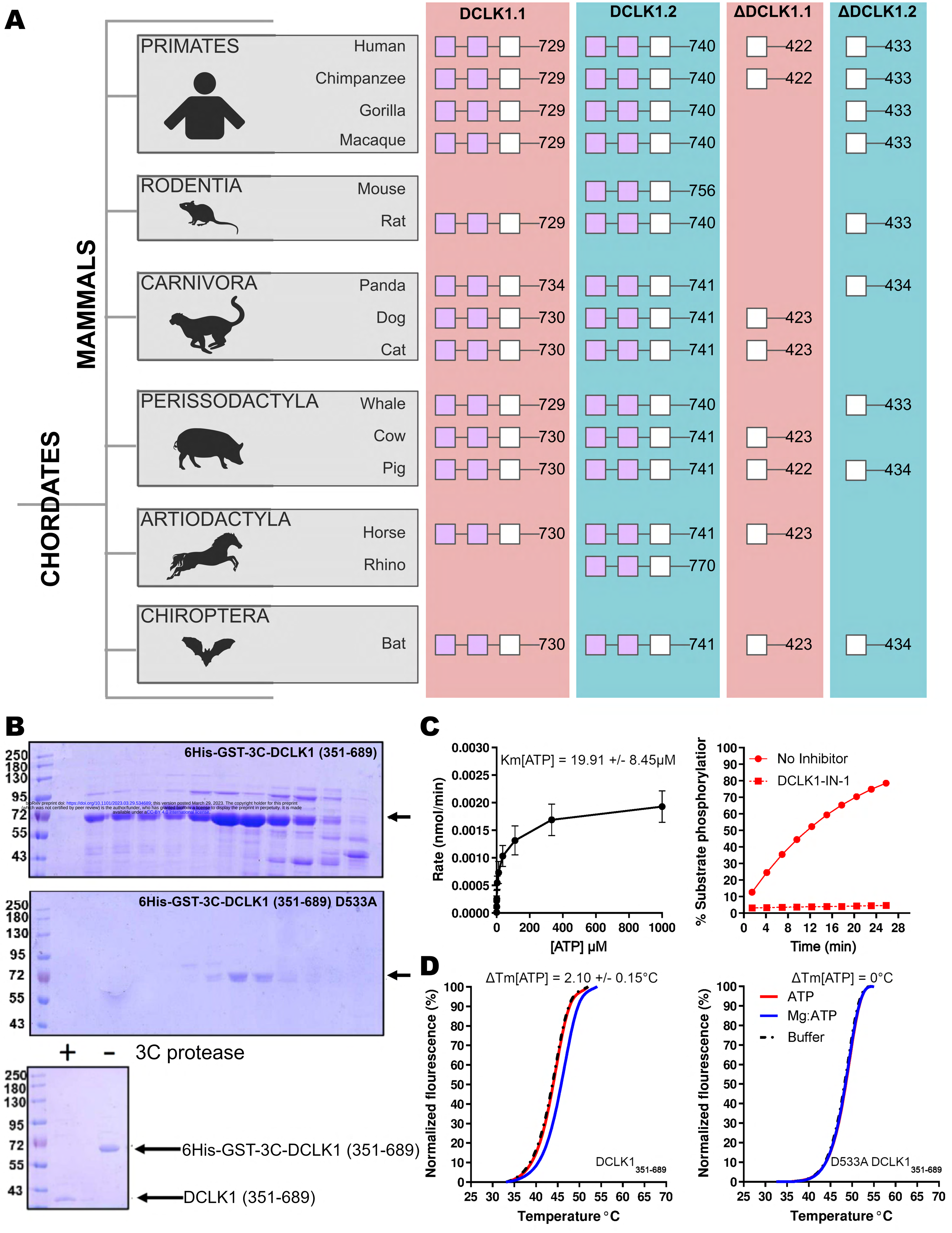

bioRxiv preprint doi: https://doi.org/10.1101/2023.03.29.534689; this version posted March 29, 2023. The copyright holder for this preprint (which was not certified by peer review) is the author/funder, who has granted bioRxiv a license to display the preprint in perpetuity. It is made available under aCC-BY 4.0 International license.

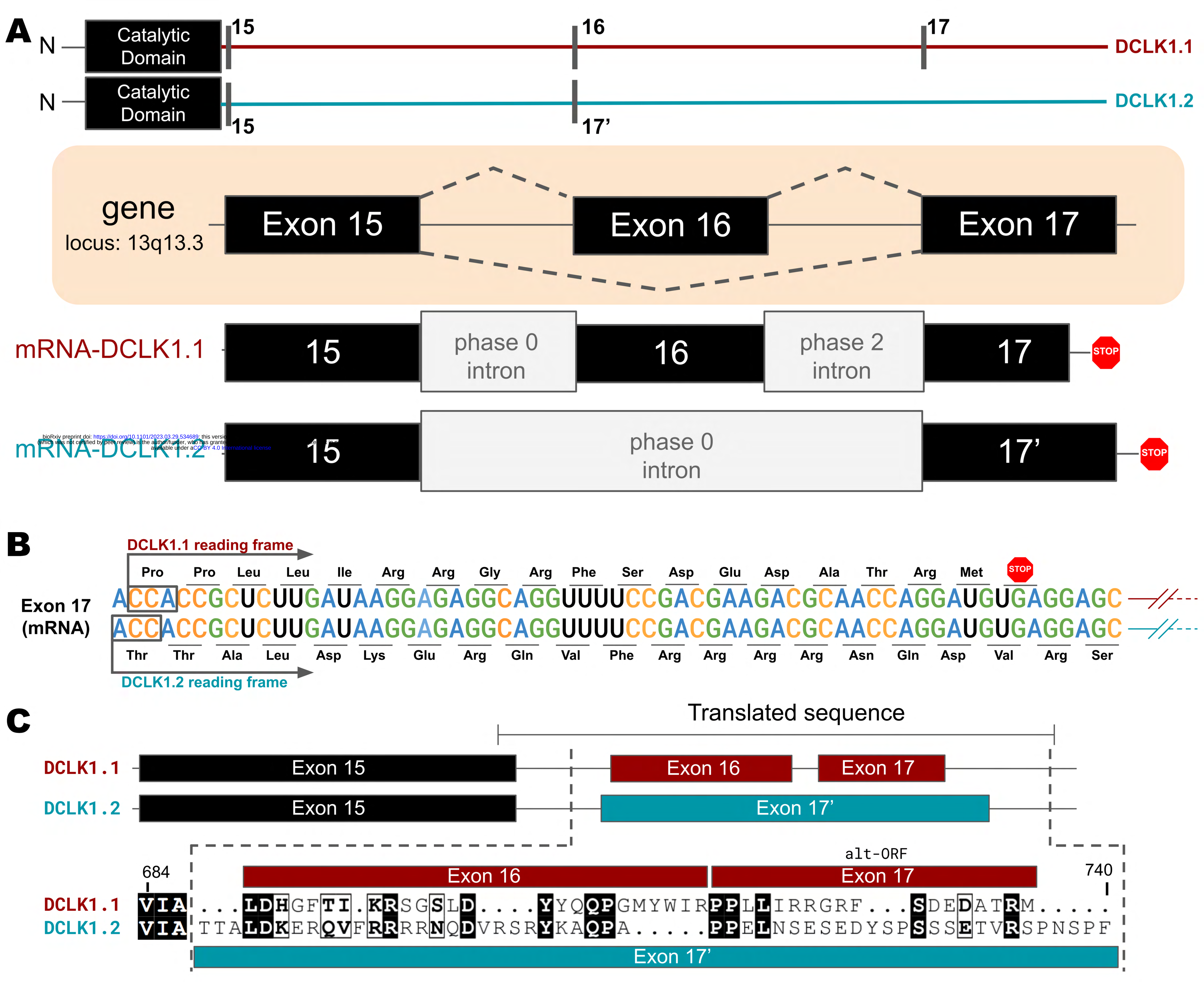

bioRxiv preprint doi: https://doi.org/10.1101/2023.03.29.534689; this versio
(which was not certified by peer review) is the author/funder, who has grante
available under aCC-BY 4.0 International license

bioRxiv preprint doi: https://doi.org/10.1101/2023.03.29.534689; this version posted March 29, 2023. The copyright holder for this preprint (which was not certified by peer review) is the author/funder, who has granted bioRxiv a license to display the preprint in perpetuity. It is made available under aCC-BY 4.0 International license.

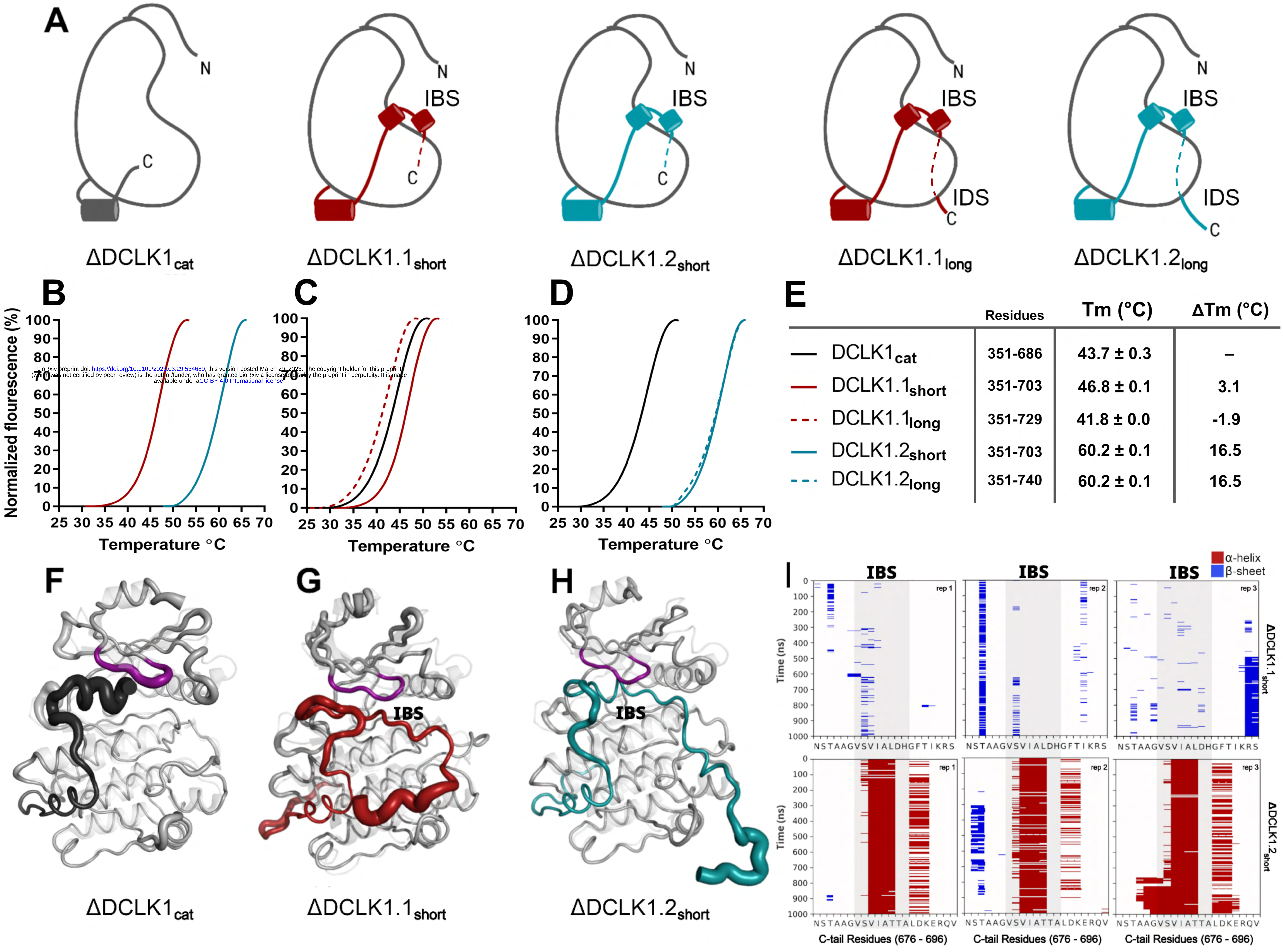

bioRxiv preprint doi: https://doi.org/10.1101/2023.03.29.534689; this version posted March 29, 2023. The copyright holder for this preprint (which was not certified by peer review) is the author/funder, who has granted bioRxiv a license to display the preprint in perpetuity. It is made available under aCC-BY 4.0 International license.

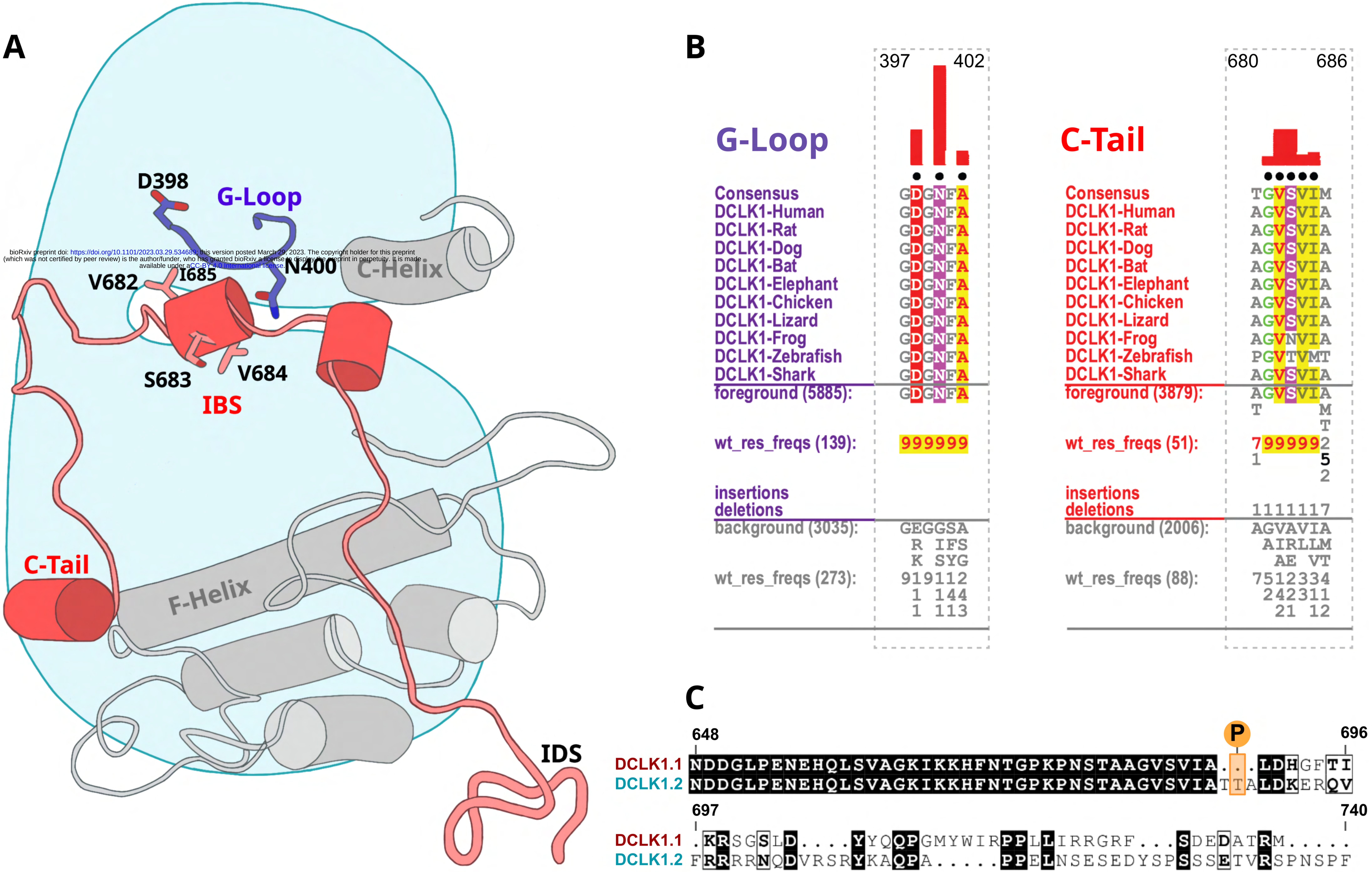

bioRxiv preprint doi: https://doi.org/10.1101/2023.03.29.534689; this version posted March 29, 2023. The copyright holder for this preprint (which was not certified by peer review) is the author/funder, who has granted bioRxiv a license to display the preprint in perpetuity. It is made available under aCC-BY 4.0 International license.

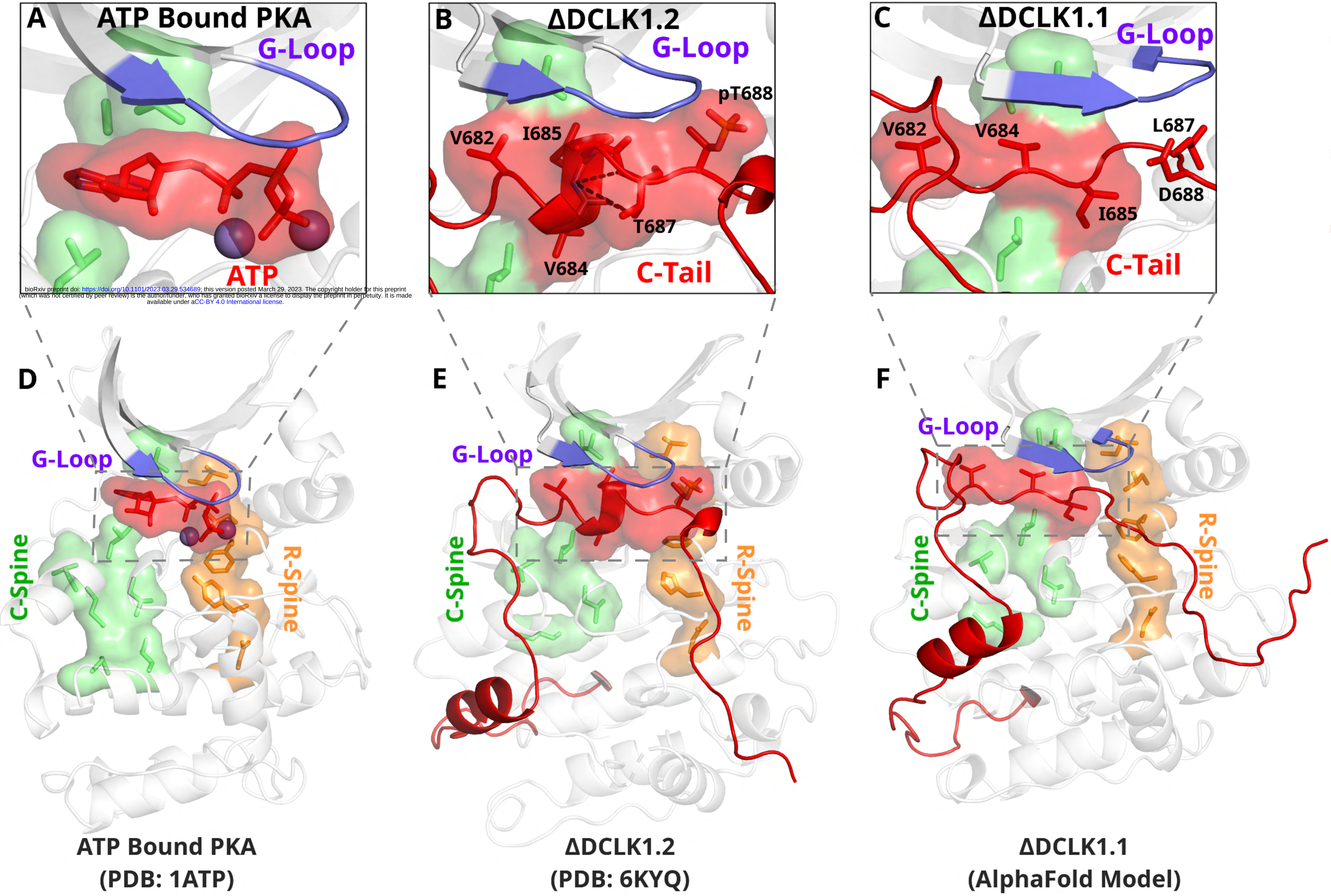

bioRxiv preprint doi: https://doi.org/10.1101/2023.03.29.534689; this version posted March 29, 2023. The copyright holder for this preprint (which was not certified by peer review) is the author/funder, who has granted bioRxiv a license to display the preprint in perpetuity. It is made available under aCC-BY 4.0 International license.

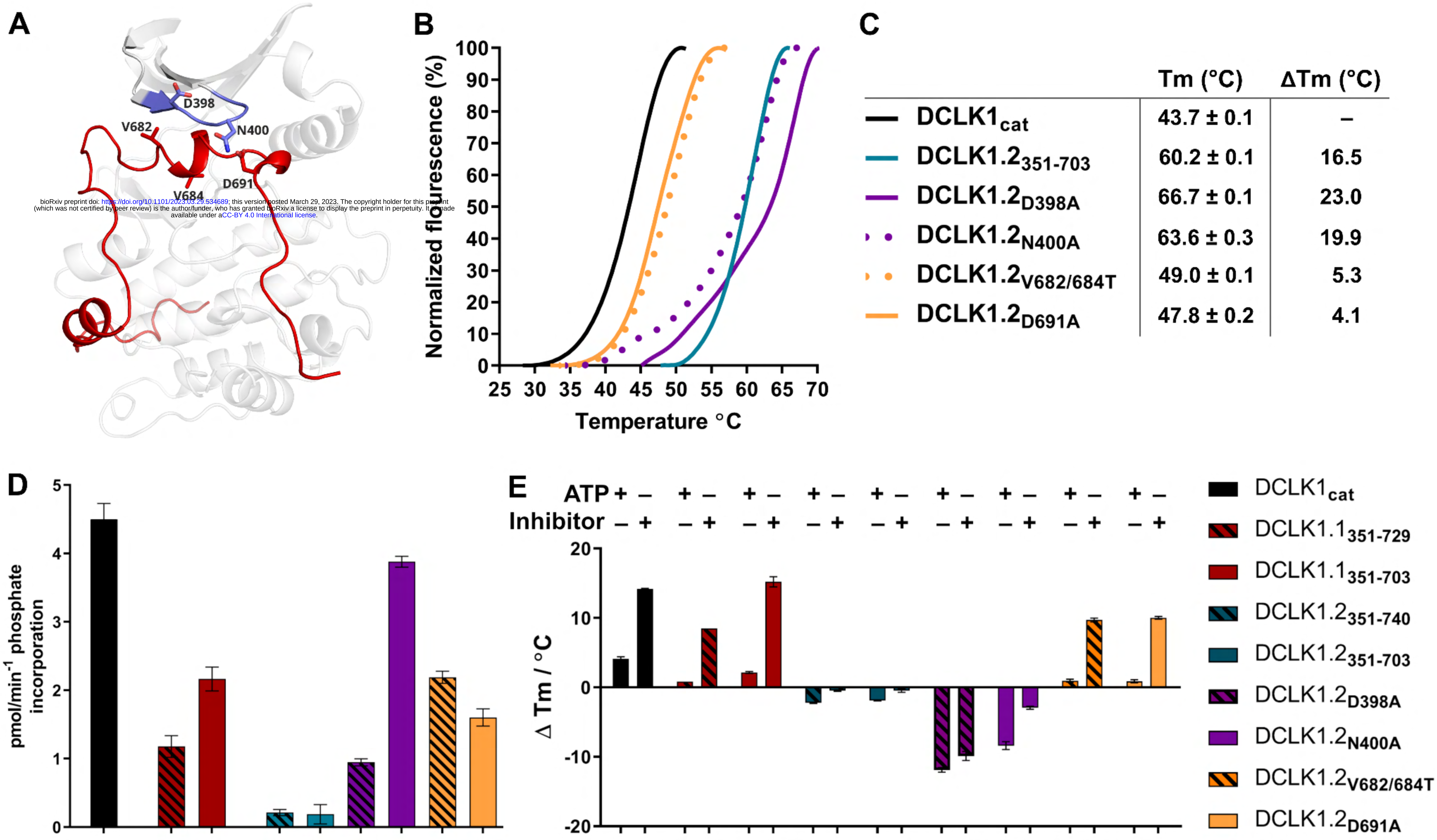

bioRxiv preprint doi: https://doi.org/10.1101/2023.03.29.534689; this version posted March 29, 2023. The copyright holder for this preprint (which was not certified by peer review) is the author/funder, who has granted bioRxiv a license to display the preprint in perpetuity. It is made available under aCC-BY 4.0 International license.

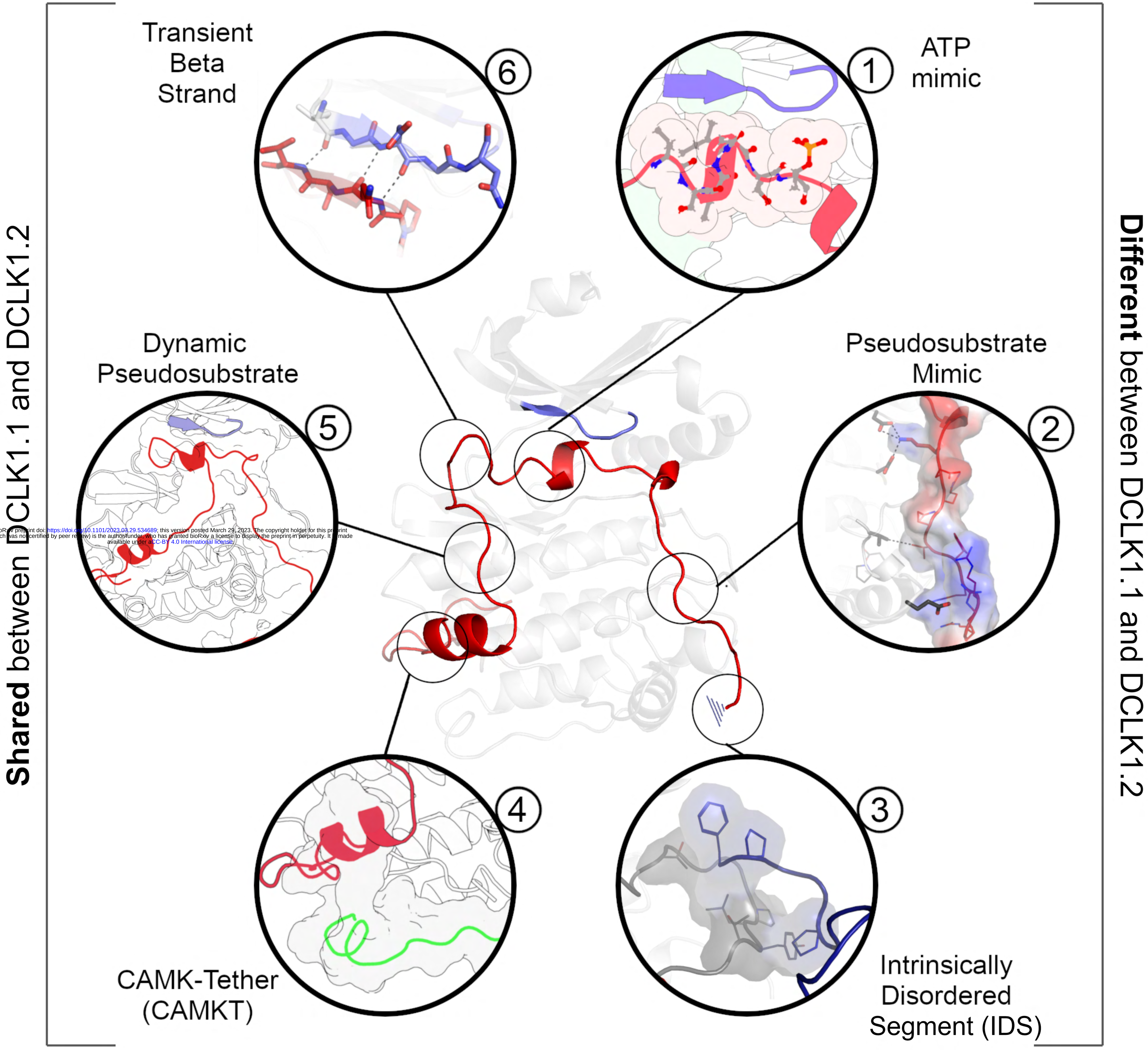

Supplement: Supplementary file 1 [file NIHPP2023.03.29.534689v1-supplement-1.pdf]
